# Heterogeneity of Lipopolysaccharide as Source of Variability in Bioassays and LPS-Binding Proteins as Remedy

**DOI:** 10.3390/ijms24098395

**Published:** 2023-05-07

**Authors:** Alexandra C. Fux, Cristiane Casonato Melo, Sara Michelini, Benjamin J. Swartzwelter, Andreas Neusch, Paola Italiani, Martin Himly

**Affiliations:** 1 Division of Allergy & Immunology, Department of Biosciences & Medical Biology, Paris Lodron University of Salzburg (PLUS), Hellbrunnerstraße 34, 5020 Salzburg, Austria; 2 Chemical Biology Department, R&D Reagents, Miltenyi Biotec B.V. & Co. KG, Friedrich-Ebert-Straße 68, 51429 Bergisch Gladbach, Germany; 3 Biotechnical Faculty, Department of Biology, University of Ljubljana, Večna pot 111, 1000 Ljubljana, Slovenia; 4 Department of Microbiology, Immunology, and Pathology, 1601 Campus Delivery, Colorado State University, Fort Collins, CO 80523, USA; 5 Experimental Medical Physics, Heinrich-Heine University Düsseldorf, Universitätsstraße 1, 40225 Düsseldorf, Germany; 6 Institute of Biochemistry and Cell Biology, Consiglio Nazionale delle Ricerche (CNR), Via P. Castellino 111, 80131 Naples, Italy; 7 Stazione Zoologica Anton Dohrn (SZN), Villa Comunale, 80121 Naples, Italy

**Keywords:** endotoxin, lipid A, immunology, detection, low endotoxin recovery, LPS-binding molecules

## Abstract

Lipopolysaccharide (LPS), also referred to as endotoxin, is the major component of Gram-negative bacteria’s outer cell wall. It is one of the main types of pathogen-associated molecular patterns (PAMPs) that are known to elicit severe immune reactions in the event of a pathogen trespassing the epithelial barrier and reaching the bloodstream. Associated symptoms include fever and septic shock, which in severe cases, might even lead to death. Thus, the detection of LPS in medical devices and injectable pharmaceuticals is of utmost importance. However, the term LPS does not describe one single molecule but a diverse class of molecules sharing one common feature: their characteristic chemical structure. Each bacterial species has its own pool of LPS molecules varying in their chemical composition and enabling the aggregation into different supramolecular structures upon release from the bacterial cell wall. As this heterogeneity has consequences for bioassays, we aim to examine the great variability of LPS molecules and their potential to form various supramolecular structures. Furthermore, we describe current LPS quantification methods and the LPS-dependent inflammatory pathway and show how LPS heterogeneity can affect them. With the intent of overcoming these challenges and moving towards a universal approach for targeting LPS, we review current studies concerning LPS-specific binders. Finally, we give perspectives for LPS research and the use of LPS-binding molecules.

## 1. Introduction

Bacteria are omnipresent in our environment, in soil, water, and in and on living organisms, such as plants, insects and animals [1]. They are also found in extreme habitats characterized by high temperatures, high and low pH values, high pressures and elevated salinity [2,3,4]. Surviving such harsh conditions forced extensive adaptation and the development of protective mechanisms. The first of these protective mechanisms is fulfilled by a complex barrier: the bacterial cell wall. On the one hand, it shields the bacterium against foreign molecules; on the other hand, it allows the selective passage of substances. Three quarters of the outer cell wall of Gram-negative (G-) bacteria are composed of lipopolysaccharide (LPS), a diverse class of amphiphilic molecules, also referred to as endotoxin due to its strong immunogenic potential. It is estimated that one single bacterium can contain more than three million LPS molecules [5]. These molecules are released into the environment during bacterial division or death. The first description of the harmful nature of LPS dates to a work from Peter Ludvig Panum (1820–1885). He stated that the ‘putrid poison’ was able to cause fever and even death when injected into dogs [6]. However, Richard Pfeiffer was the first to use the term endotoxin for this class of natural molecules. Pfeiffer brought along the first evidence for the toxin’s heat stability and difficult inactivation, both of which have remained a major issue to this day [7].

Since bacteria are omnipresent in the environment, an immense and constant load of LPS molecules is released into our surroundings, which becomes especially dangerous during an injury or a bacterial infection, when these organisms transpose our epithelial barrier. High levels of LPS in the blood can cause fever, septic shock, and eventually death. Accordingly, contact with LPS-contaminated vaccines, drugs, and medical devices that penetrate the skin can expose the human immune system to endotoxin, and hence trigger these symptoms. To guarantee the safety of pharmaceuticals and medical devices, regulatory agencies strictly define upper limits for LPS in medicinal products. Additionally, guidelines define which quantification methods for LPS are accepted [8]. The established LPS quantification methods strongly rely on ideal conditions (e.g., lack of interferences, dilutions, and specificity), and often detect LPS reliably only from certain bacterial strains [9].

**Figure 1:**
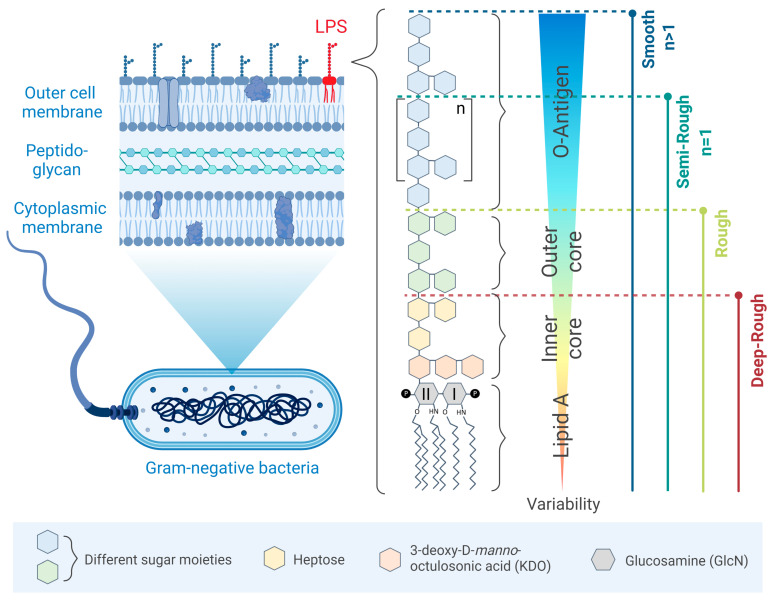
Schematic illustration of the LPS structure. LPS shown in bright red is anchored in the outer membrane of (G-) bacteria. Smooth- (S), semi-rough- (SR), rough- (R) or deep-rough- (DR) LPS types are defined by the length of the O-antigen and core region. The variability decreases from the outermost part of LPS to the hydrophobic innermost part. Different sugar moieties are shown in blue and green; heptose is shown in yellow; 3-deoxy-D-*manno*-octulosonic acid (KDO) in orange, and glucosamine (I and II) in gray.

Part of the observed variability in detection and quantification between distinct bacterial strains resides in the chemical heterogeneity of LPS, which differs within its different moieties as well. The most conserved moiety of LPS is its hydrophobic part, known as lipid A, which is anchored to the outer membrane and contains several acyl chains. In contrast, the most variable region is the hydrophilic part of LPS, which is localized facing the environment and is called O-antigen (Figure 1). Bacteria with an O-antigen within their LPS structure are classified as smooth (S) strains. If this part is lacking, the bacteria are categorized as rough (R) strains. Bacteria with only one O-chain repeating unit are referred to as semi-rough (SR) strains [10]. It was shown that absence of O-antigen in the outer membrane decreases its stability, and hence increases stress levels for the bacterium [11].

**Figure 2:**
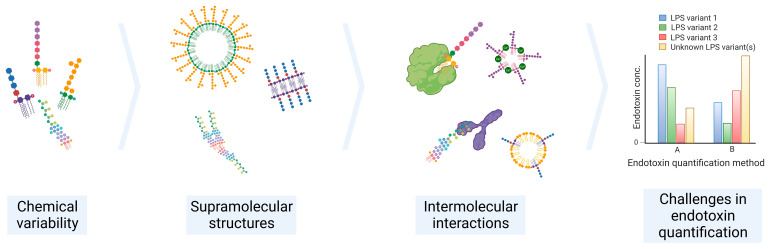
Overview of the main challenges in endotoxin quantification. The chemical variability of LPS, supramolecular structures, and interaction with molecules result in inaccurate and/or unreproducible endotoxin quantification measurements.

The connecting region between the O-antigen and lipid A part is denoted as the core region of LPS. It establishes this linkage via two glucosamines (GlcN). The number and length of the acyl chains in the lipid A part defines the biological activity and the proinflammatory potential of the molecule once exposed to the human immune system. Any modification in the chemical structure can drastically decrease the biological activity [12]. The impact of lipid A’s heterogeneity on the immune response will be elaborated on in more detail in Chapter 3. The modification of the chemical structure of LPS together with the environmental conditions (e.g., pH, temperature, salt concentration, and proteins) may change the supramolecular structure of LPS, and, consequently, the interaction with other molecules and LPS sensors, making its detection and quantification a challenging process (Figure 2).

In this review, we first comprehensively overview LPS heterogeneity, in particular the great variability between lipid A moieties highlighting their composition divergences and the formation of supramolecular structures. We then establish a connection with the ways in which these differences affect the immune system. Afterwards, we discuss the impact of current quantification methods, as well as the phenomenon called low endotoxin recovery (LER). Finally, we focus on the connection of LPS heterogeneity and known LPS-binding molecules. Here, we describe the perspectives they pose for future LPS research and applications towards universal detection, quantification, and inactivation approaches.

## 2. LPS Heterogeneity

### 2.1. Chemical Heterogeneity of LPS

The (G-) bacterial cell wall’s main function is to protect the microorganism from environmental factors such as antibiotics and environmental stresses. Additionally, it facilitates resistance against bactericidal agents by enabling the evasion from the host immune system or reducing membrane permeability for antimicrobial peptides. Since LPS is part of the outer membrane, it directly interacts with its surroundings. Adaptation to harsh environmental conditions induces changes in the bacterial metabolism, including LPS biosynthesis, and therefore, the chemical structure of single LPS components [13]. As previously mentioned, the three chemical moieties of LPS are the O-antigen, the core, and lipid A. The O-antigen consists of a variety of sugar moieties, including a repetitive polysaccharide monomeric unit that can build a chain of up to 50 repeats. If the amount of saccharides is reduced in the polymeric chain, this truncated version of LPS is referred to as lipooligosaccharide (LOS) [14]. Due to its uniqueness, the O-antigen serves as a fingerprint to determine bacterial species and serotype [15]. The number of saccharide units of the O-antigen is highly variable not only throughout different species but also within one single bacterium. Smooth types ((G-) bacteria comprising an O-Antigen) are more commonly found in nature. Under certain conditions, smooth strain bacteria can mutate to rough strains (lacking O-Antigen) to omit the energy-intensive synthesis of O-antigen. This transition is, most of the time, irreversible [16]. Pupo and co-workers isolated and separated smooth, semi-rough, and rough LPS variants from the same *Escherichia coli* O111 wildtype by gel electrophoresis. By checking their biological activity, they found that rough types dominated the activation of human macrophages when compared to smooth types [17].

**Figure A1:**
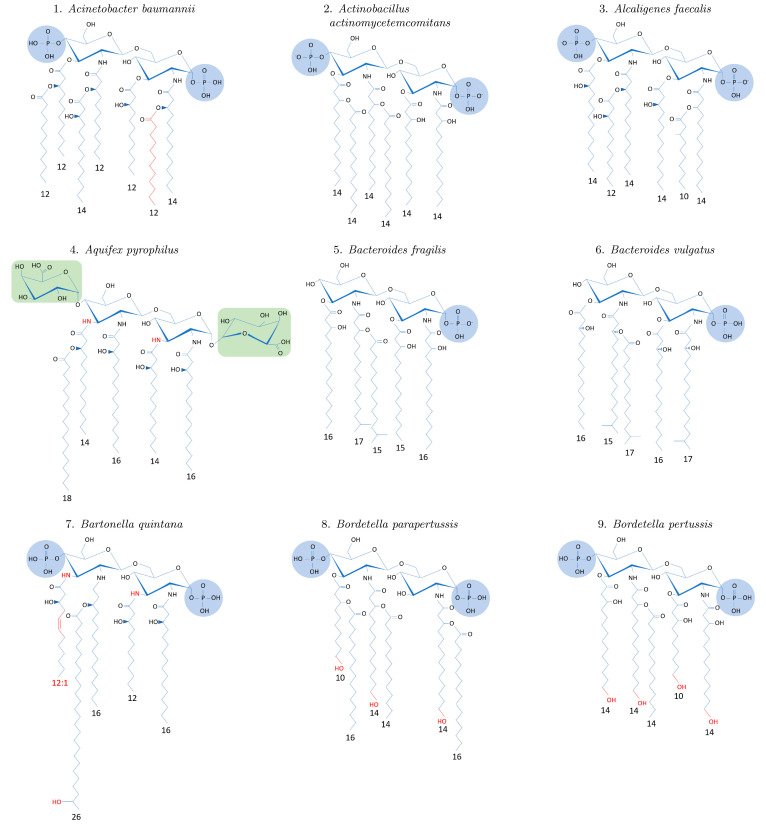

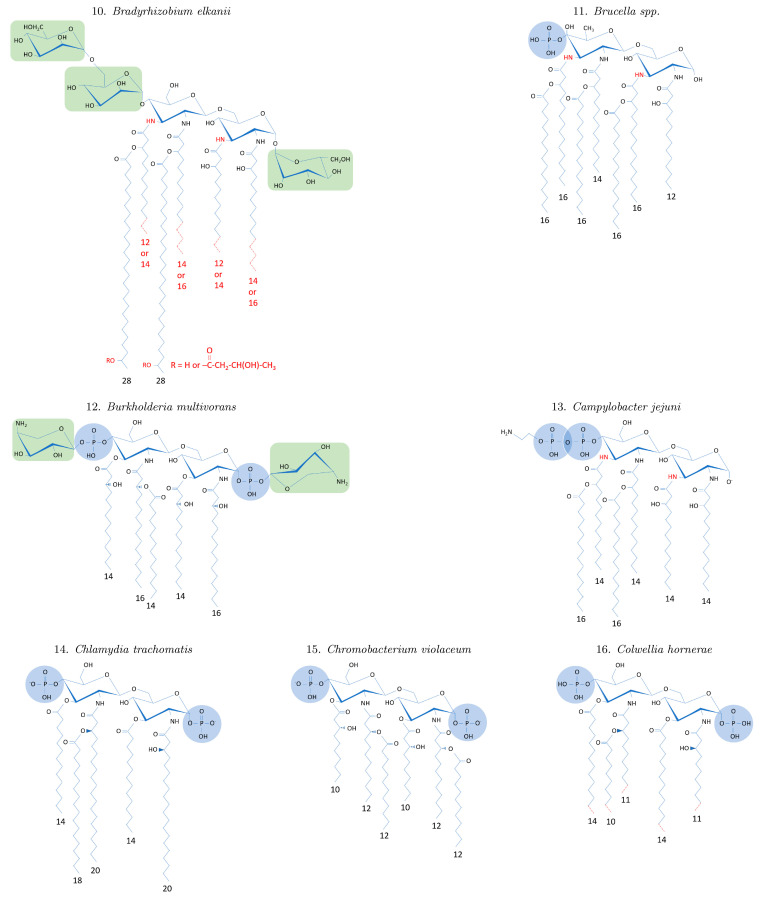

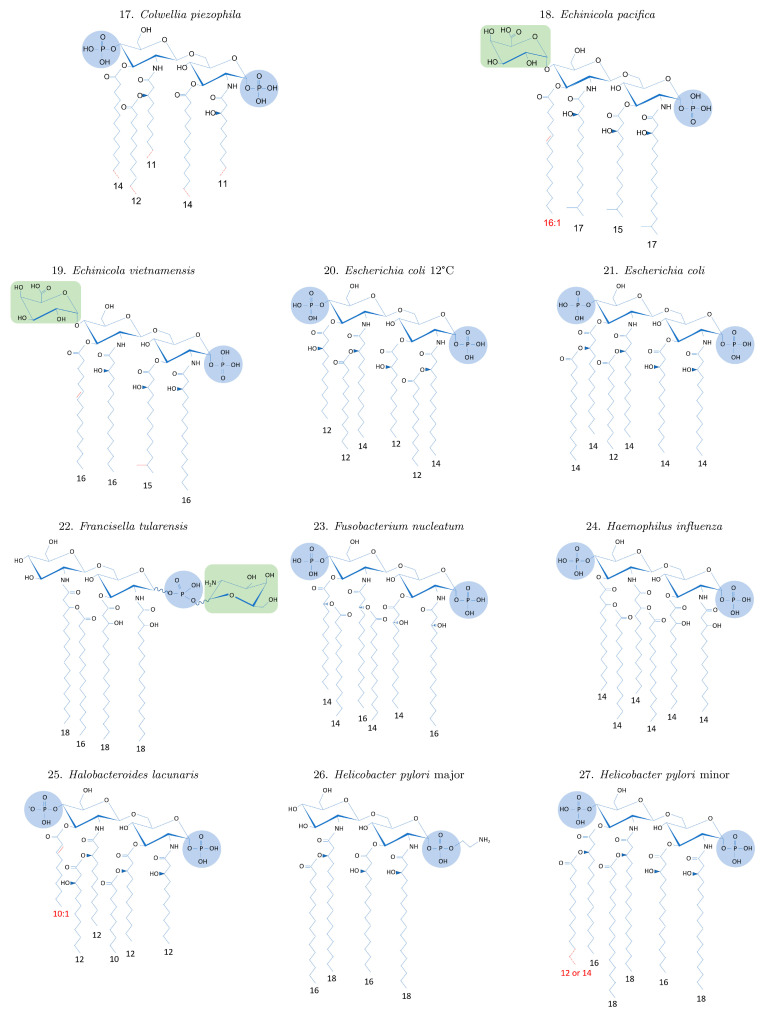

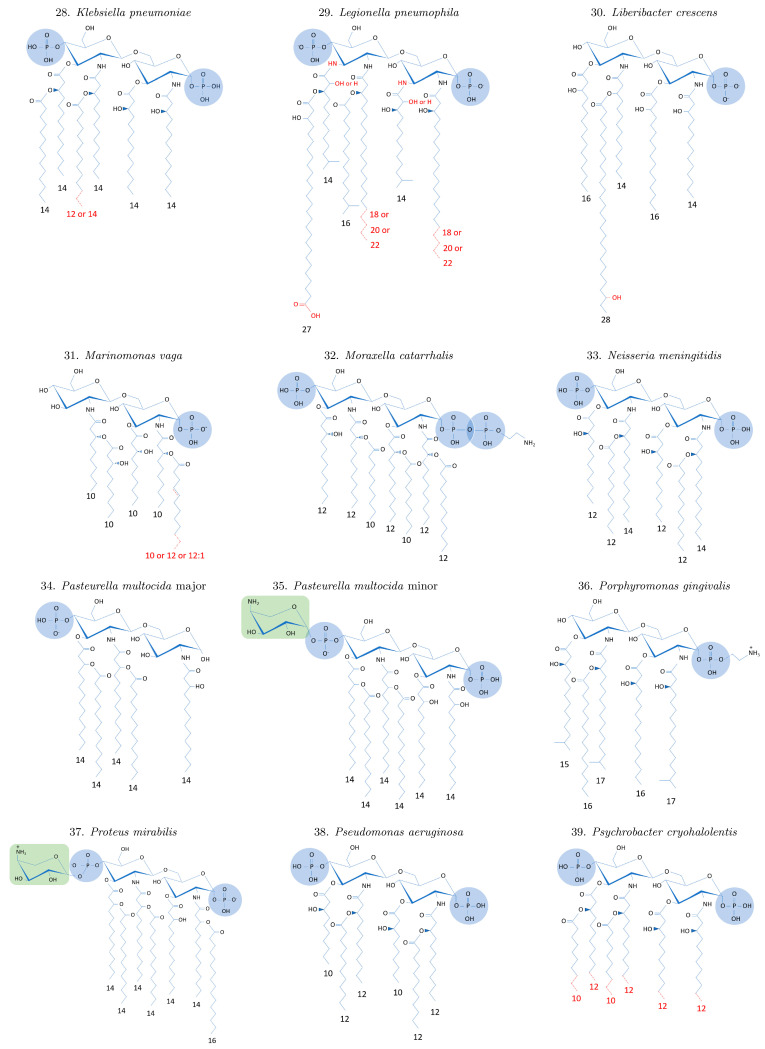

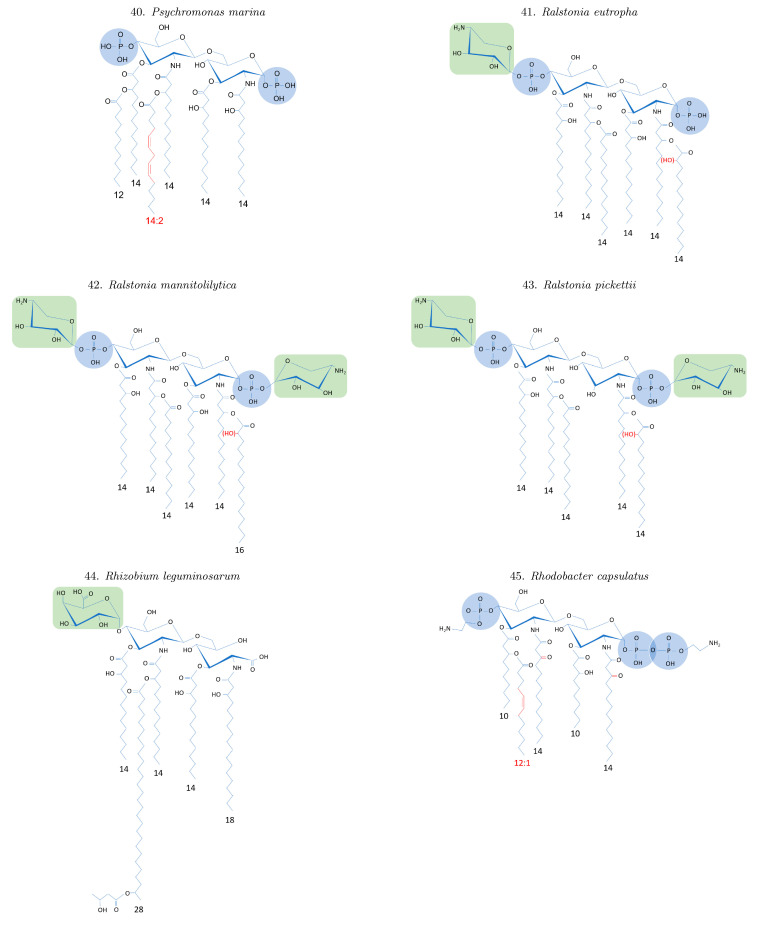

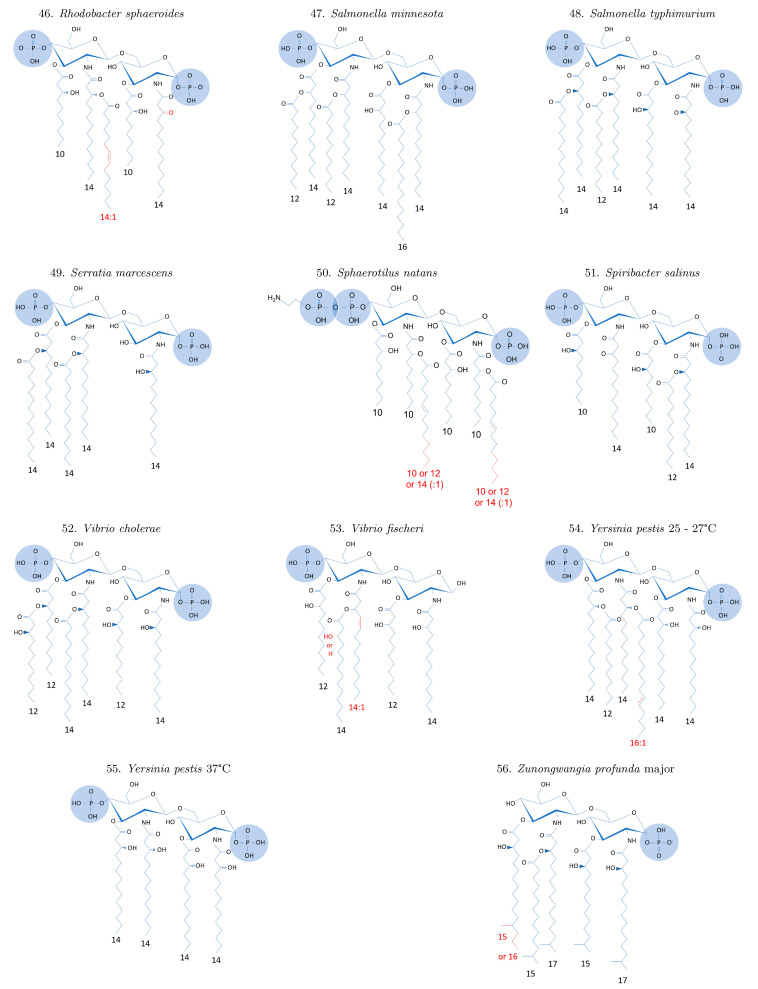
Structures of lipid A from different bacterial species. References to structures are listed in Table 1. Possible variations of the structures are marked in red.

In rough strain bacteria, the LPS core is directly exposed to the bacterial outer environment. The core is less variable than the O-antigen and consists only of a few sugar moieties. It is divided into two parts: the inner and the outer core. Strains that lack the O-antigen and the outer core are further categorized as deep-rough (DR) strains (Figure 1). The outer core typically comprises the hexoses glucose, galactose, and N- acetylglucosamine, among others [18]. In *Escherichia coli*, five different core types are known; they share a (hexose)_3_ carbohydrate backbone and two side chain residues in this backbone [19]. The inner core is less variable than the outer core and contains the sugar 3-deoxy-D-*manno*-octulosonic acid (KDO). KDO serves as the linker between the LPS core and its most conserved and hydrophobic region: lipid A. The backbone of lipid A consists of two sugar rings (I and II) that form a D-glucosamine (GlcN) disaccharide. The backbone is connected to the inner core through GlcN II. The sugar dimer GlcN can appear unmodified, mono- or di-phosphorylated at C1 for GlcN I and C4 for GlcN II, respectively. Among others, *Escherichia coli*, *Serratia marcescens*, and *Neisseria meningitidis* are endued with two phosphate groups, while *Sphaerotilus natans*, *Moraxella catarrhalis*, and *Rhodobacter capsulatus* show a tri-phosphorylated GlcN dimer [20,21,22]. Mono phosphorylation can occur on either of the two sites. For instance, *Porphyromonas gingivalis* and *Bacteroides vulgatus* are phosphorylated on GlcN I, whereas at the same time GlcN II is hydroxylated at C4. However, these positions can also be occupied by sugar moieties [23,24,25]. The marine species *Echinicola pacifica* and *Echinicola vietnamensis* possess a D-galacturonic acid (D-GalA) at the C4 position [26]. In addition to the phosphorylated examples, species that come without phosphorylation have been discovered. One example is *Aquifex pyrophilus* which possesses two GalA instead of phosphates [23]. The fatty acids of lipid A are linked to GlcN by an ester at C2 and amine residue at C3. However, in some species, such as *Legionella pneumophila*, the amine linkage is replaced by a second amine [27]. Changing the overall molecular charge by the loss or addition of phosphate groups and/or the addition of positively charged sugar units can influence the resistance against cationic antimicrobial molecules [28,29]. The hydrophobic acyl chains of lipid A are commonly bound to the GlcN by two ester and two amide bondings, while some species form only amide bondings [23,30]. These acyl chains vary amongst species by the number of attached chains to the two GlcN as well as their length (Figure A1). These fatty acids are anchored in the lipid layer of the outer membrane and play a role in the growth and survival of the bacterium [31]. Structural differences of lipid A—for instance, the symmetric arrangement and status of phosphorylation—are summarized in Table 1. Depending on the arrangement of the acyl chains on each GlcN, it appears in a symmetrical or asymmetrical shape. Asymmetric strains possess unequal amounts of chains on each GlcN, as for instance, *Escherichia coli* with an arrangement of 4 + 2, as well as *Porphyromonas gingivalis* with a 3 + 2 arrangement. *Neisseria meningitidis* is known to be symmetrical with three acyl chains on each GlcN. The structure of the acyl chains is prone to modifications as well. Besides additional keto or hydroxyl groups or unsaturated carbon bonds in the center of the chain [32,33], branching and terminal hydroxyl groups [34] are occasionally seen. Mass spectrometry analysis revealed that one species often implicates a mixture of lipid A compositions. For instance, *Pasteurella multocida* was found to have different lipid A versions ranging from tetra- penta-, hexa- to hepta-acylation, among which the penta-acylated lipid A is the most abundant [35]. The lipid A structure that is reported to have the strongest endotoxic effect is the di-phosphorylated asymmetric hexa- (4 + 2) form that is encountered in strains of *Escherichia coli* and *Salmonella enterica* [36], as elaborated further in Chapter 3. Despite being the most conserved part of LPS, there is great variability between lipid A chemical structures. These alterations can be caused, among others, by environmental influences, such as high pressure [37], higher saline levels in the environment [38], and/or change in temperature [39,40,41]. Marine bacteria often face one or more of these extreme habitats, such as a thermal vent in the deep sea. A high intra-species heterogeneity was observed for the recently discovered strain *Zunongwangia profunda* SM-A87 [42] and different psychrophilic bacteria isolated from Antarctica [39]. Furthermore, some lipid A strains only show four acyl chains, a mono-phosphorylated backbone, and an additional D-galacturonic acid, e.g., found in *Echinicola pacifica* and *Echinicola vietnamensis* [26]. This again highlights the variability and options for the structural changes of lipid A, as these changes cannot only be caused by low or elevated temperatures [41] but also by altering other growth conditions, such as osmolarity [43] or pH [44,45].

### 2.2. LPS Supramolecular Structures and Intermolecular Interactions

The complexity of LPS is not restricted to its intrinsic difference in the sugar composition of the polysaccharide part or lipid A, it also derives from the supramolecular aggregation states [80]. The amphiphilic nature of LPS plays a role in the formation of these aggregates. In general, amphiphilic molecules can be encountered in their monomeric form in diluted solutions; however, when the critical micellar concentration (CMC) is reached, the molecules tend to aggregate and form micelles. In fact, the determination of CMC is dependent on several physical properties, such as osmotic pressure, turbidity, electrical conductance, and surface tension [81]. In the context of LPS, the molecular weight of the monomeric form can vary from 2.5 kDa (R-form) to 70 kDa (S-forms with long O-antigens), being the average between 10 and 20 kDa [5,80,82]. During LPS aggregation, monomers are believed to interact through the non-polar attraction between the lipidic chains and electrostatic bridges among the phosphate groups and divalent cations present in the solution [80]. Here, the CMC, size and shape of aggregates differ mainly depending on the chemical structure of LPS, pH, temperature, amount and type of ions in solution and presence of surfactants, proteins or other molecules [80,83,84]. Smooth strains tend to form aggregates at higher LPS concentrations than rough strains. This characteristic can be explained by the higher hydrophobicity of the rough strains, lacking the hydrophilic O-antigen region [83]. Additionally, it is expected that simple surfactants, such as SDS, aggregate cooperatively and exhibit a narrow CMC, whereas the determination of the CMC for amphiphilic molecules with a broader molecular weight distribution is rather complex [83]. CMCs ranged from 10 to 14 µg/mL with the presence of pre-micelles or evolving micelles until higher concentrations were reported. [81,83,85]. Micelles and other LPS supramolecular structures were observed using different techniques, such as electron micrograph, dynamic light scattering, small angle neutron scattering, cryo-transmission electron microscopy, synchrotron small-angle X-ray scattering, fluorescence correlation spectroscopy, and infrared spectroscopy [81,85,86,87,88,89].

Furthermore, the supramolecular structures of LPS can be altered by intermolecular interactions [88,90]. As expected, several proteins that interact with LPS are known to be involved in immunological responses. For instance, LPS interacts with classical biological molecules, such as the lipopolysaccharide-binding protein (LBP), the bactericidal permeability-increasing protein (BPI), factor C from the limulus amebocyte lysate (LAL) cascade reaction, cluster of differentiation 14 (CD14), CD16, CD18, and antibodies. However, besides those proteins, other positively charged proteins are likely to electrostatically interact with LPS (e.g., lysozymes and lactoferrin) [80,91]. Interestingly, it is also possible to find neutral proteins (e.g., hemoglobin) and even negatively charged proteins (e.g., BSA) interacting with LPS [92,93]. The mechanisms behind the interaction between LPS and negative and neutral proteins is still not completely clarified. Nonetheless, there are some possible explanations. The first is the hydrophobic interaction between the protein and lipid A. A second possibility is that the protein’s carboxyl and the phosphate groups of LPS might compete for dications (e.g., Ca^2+^), forming a dynamic calcium bridge between protein and LPS [80]. LPS interactions with peptides were studied by Hong and coauthors, who observed conformational changes in LPS self-assemblies, changing from micelles to multilamellar structures, upon addition of increasing concentrations of the antimicrobial peptide (AMP) human cathelicidin (LL-37) to LPS solutions [88]. Studying the same antimicrobial peptide, Bello et al. observed changes in LPS conformation after the addition of LL-37 to a high concentration of (S) type LPS from *Escherichia coli* O111:B4 in the presence of dication Mg^2+^. While LPS alone formed elongated micelles, branched structures, and toroids, the addition of LL-37 prevented the connection between these structures and the formation of toroids. The authors also tested (R) strains, which presented irregular lamellae or sheet-like structures coexisting with toroids [87,94]. The interaction of LPS with bile salt sodium deoxycholate was studied by Ribi et al., who described that upon interaction, LPS dissociated into subunits with molecular weights around 20 kDa that are not toxic for rabbits. However, after dialysis, the endotoxin reaggregated into a biologically active form with sizes ranging from 500 to 1000 kDa [86]. Hence, recognition of LPS by the immune system, as well as the detection by standard endotoxin quantification assays can be influenced by the supramolecular structure.

Likewise, as described, the supramolecular structure of LPS cannot be reduced to a defined micelle, but different shapes and sizes of aggregates can be formed depending on the chemical structure of LPS and the environmental conditions. This characteristic has significance for the safety aspects of the immunological and pharmaceutical fields, where it is of utmost importance to handle phenomena such as the low endotoxin recovery effect (LER). In this case, measurements result in a lower quantity even in cases when a known amount of endotoxin is added to a sample (see Chapter 4.2). It is also relevant for the understanding of how anti-microbial molecules interact with (G-) bacteria, and it plays a role in the design of new drugs against these microorganisms [88,90].

## 3. LPS Recognition and Immunological Impact

**Figure 3:**
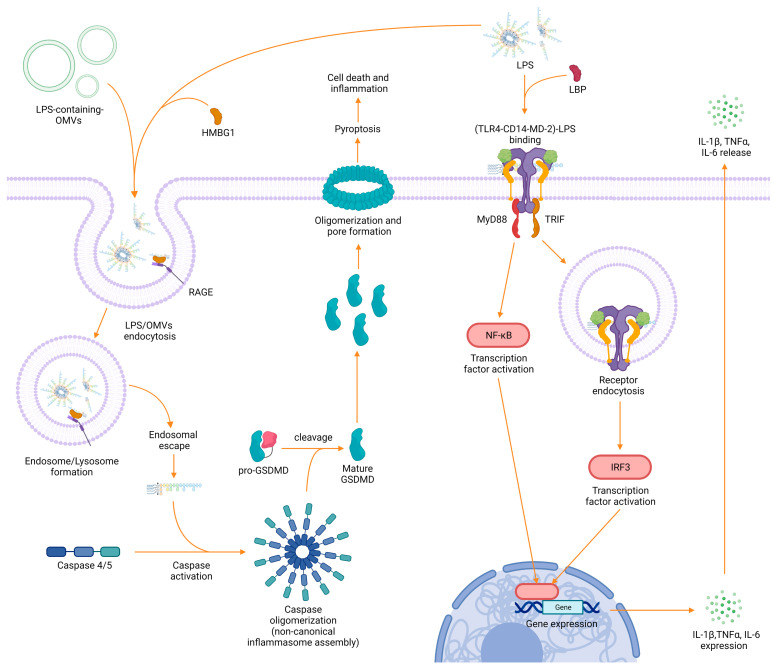
Scheme of the most relevant pathways for LPS detection in human cells. Left: detection of intracellular LPS via caspase proteins causes the assembly of a supramolecular complex called inflammasome, which induces the initiation of the potent inflammatory process called pyroptosis. Right: detection of extracellular LPS via the TLR4 receptor complex and initiation of inflammatory responses via the MyD88 and TRIF signaling cascades.

As LPS is a ubiquitous component of the outer membrane of (G-) bacteria, it is one of the key targets by which a host may detect and respond to a bacterial invasion. The immunological consequences after exposure to LPS do not only depend on the source and the supramolecular structure of LPS, but also on the signaling pathways that are stimulated. The most relevant innate immune receptor activated by extracellular LPS is toll–like receptor 4 (TLR4), which was identified in the late 1990s [95,96]. TLR4 is mainly expressed on the surface of both immune cells—such as monocytes, macrophages, neutrophils, dendritic cells, and natural killer cells [97,98]—and somatic cells—including fibroblasts and epithelial cells [97,99]. The signaling pathway that leads to TLR4 activation is initiated when extracellular LPS is perceived via its lipid A moiety by soluble LBP. LBP then delivers LPS to the membrane accessory protein CD14, which in turn transfers the bound molecule to the myeloid differentiation factor 2 (MD-2). The loaded MD-2 is then recognized by TLR4 promoting the assembly of a supramolecular TLR4-CD14-MD-2 receptor complex [100,101]. The formation of this complex initiates an intracellular signaling cascade, mediated by the adaptor proteins myeloid differentiation primary response 88 (MyD88) and TIR-domain-containing adapter-inducing interferon-*β* (TRIF), which leads first to the activation of the transcription factors interferon regulatory factor 3 (IRF-3) and nuclear factor *κ*-light-chain-enhancer of activated B cells (NF-*κ*B), and then to the production of potent inflammatory proteins, such as the cytokines IL-1*β*, TNF-α, IFNs, and IL-6 (Figure 3) [102,103,104,105].

**Figure 4:**
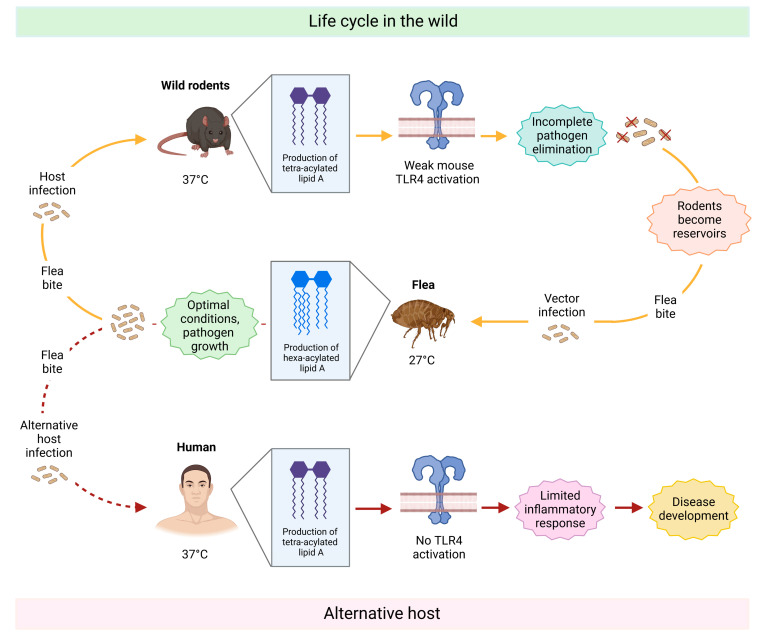
Schematic representation of *Yersinia pestis* life cycle with focus on how the lipid A moiety changes during the different stages, ultimately interfering in the host’s immune response. The top part of the image shows the life cycle in wild animals. Briefly, *Yersinia pestis*-infected fleas bite wild rodents and transmit the pathogen. Triggered by the rodent’s body temperature of 37 °C, it starts producing a less immunogenic variant of lipid A (tetra-acylated), which is not able to elicit an effective immune response. As a result, *Yersinia pestis* is not fully eliminated and persists. The rodent becomes a reservoir of the pathogen in the wild and can, in turn, infect vector fleas, which feed on their blood. Once *Yersinia pestis* reaches the flea digestive tract (27 °C), it produces a hexa-acylated form of lipid A and starts proliferation in these favored conditions. By biting a new host, the flea can further spread the disease. Once humans are accidentally bitten (bottom part of the figure), the bacterium produces the tetra-acylated form of lipid A again to adapt to the human body temperature. However, in contrast to rodent TLR4, the human TLR4 does not recognize this lipid A variant, and therefore, it is not able to activate the downstream signaling cascade. As a result, the immune response is ineffective as it has to rely only on other defense mechanisms. *Yersinia pestis* invades the body undisturbed, causing a disease called plague.

It has recently become clear that LPS may also access the cytosol via the clathrin-mediated endocytosis of outer membrane vesicles (OMVs) [106,107] released from bacteria during growth and intracellular infections [107,108,109]. Another access pathway might be through the high-mobility group box 1 (HMGB1) protein, which can bind LPS extracellularly and favor its uptake and subsequently its endo-lysosomal escape in conjunction with the receptor for advanced glycation end products (RAGE) [110,111]. Once in the cytosol, LPS can be bound with great affinity by the caspase activation and recruitment domain (CARD) of proteins known as caspases 4 and 5. Upon binding, these zymogens undergo an autoproteolytic processing that converts them to their bioactive form, which in turn triggers the highly inflammatory process of cell death known as pyroptosis. During pyroptosis, caspases oligomerize to form inflammasomes, which in turn cleave the pro-protein gasdermin D (GSDMD), leading to its maturation. Mature GSDMD then migrates to the membrane to form large transmembrane pores that cause cell swelling and lysis, ultimately favoring the release of highly inflammatory intracellular content in the cell surrounding and destroying the replication niche of intracellular bacteria (Figure 3) [112]. Further LPS-sensing mechanisms and binding molecules have been identified in recent years, including lactoferrin, beta-defensins, and the transient receptor potential cation channel, subfamily A, member 1 (TRPA1) [113], which plays a crucial role in neurogenic inflammation and pain production during infection with (G-) bacteria [113,114,115,116]. Excessive exposure to LPS and subsequent activation and maturation of immune regulatory proteins, especially TLR4 and caspases, can potentially lead to fatal endotoxin shock [117]. However, in nature, the overall role of LPS-sensing molecules is intended to be protective, with the immunological purpose being the elimination of harmful pathogens. The magnitude of immune response following LPS detection is thus a crucial issue for healthy host defense, with LPS heterogeneity being a key determinant to this end. An important component leading to LPS recognition is the O-antigen. As the externally exposed molecule from the bacterial cell-wall, it is the first moiety responsible for interaction with the immune system, and therefore, the first indication of insult against host molecules. However, Duerr et al. found that wild-type *Salmonella* strains with an intact O-antigen delayed TLR4-mediated immune activation compared to isogenic O-antigen-lacking mutants in the gut, facilitating bacterial survival and proliferation in the host [118]. A similar delaying effect was observed in the innate immune response of plants when attacked by the plant pathogen *Xylella fastidiosa* [119]. Moreover, in the last 25 years, it has become increasingly clear that in the context of innate immune reactivity, all LPS molecules are not created equal; in fact, modifications to the lipid A chemical structure can dramatically impact the potency of TLR4 activation [12]. LPS strains that activate the TLR4-mediated pathway are called agonists (e.g., lipid A from *Escherichia coli*, *Salmonella typhimurium*, and *Klebsiella pneumoniae)* and can induce secretion of potent pro-inflammatory cytokines, such as IL-6, TNF-α and IL-1β, which if not under control can cause a lethal endotoxin shock [120]. In contrast, LPS molecules that are only able to activate the TLR4 receptor cascade in a milder level, due to changes in the lipid A chemical structure, are called weak agonists (e.g., lipid A from *Alcaligenes faecalis*, *Bacteroides vulgatus*, and *Bordetella parapertussis)* [48,50,121]. Overall, despite the lower variability in lipid A chemical composition between different bacterial species compared to the O-antigen, hexa-acylated (with acyl chains of 12–14 carbon atoms) and di-phosphorylated, lipid A molecules were found to be among the most potent activators of TLR4, TRPA1, and intracellular caspases [12,109,113,122,123,124]. Caspases, however, seem to respond to lipid A variants more broadly and with less sensitivity toward structure than does the TLR4 receptor complex [109,125]. Recent experiments have found that the acyl chain length in particular is a key component that determines the overall potency of immune response following LPS detection [126]. Besides the immune-stimulatory agonistic and the weakly agonistic variants, there are LPS molecules that can act as antagonists. Antagonistic LPS variants do not activate the immune system and can even block LPS-binding sites (e.g., TLR4 receptors), preventing the binding of agonistic LPS variants and inhibiting the downstream inflammatory cascade [127]. They are mainly tetra-acylated, present longer acyl chains, often lack one or more phosphate moieties and are found in bacterial species such as *Chlamydia trachomatis*, *Halobacteroides lacunaris*, *Bartonella quintana* (penta-acylated), *Rhodobacter sphaeroides*, *Rhodobacter capsulatus*, *and Bradyrhyzobium* strains (presence of very long-chain fatty acids (VLCFA)) [51,62,109,127,128] (see also lipid A structures in Figure A1). Lembo-Fazio et al. found that induction of cytokines in HEK293 TLR4/MD2-CD14-transfected cells was potent under hexa-acylated *Escherichia coli* LPS stimulation, but significantly reduced upon co-incubation with LPS from *Bradyrhyzobium*. The ability of lipid A from *Bradyrhyzobium* to destabilize the MD2-TLR4 complex and reduce the secretion of cytokines is suggestive of its immune antagonism [129]. Interestingly, in some rare cases, the same LPS variant can act as an agonist, as well as an antagonist, depending on the host. This phenomenon was observed for the LPS precursor lipid IVa, which activated mouse macrophages but acted as an antagonist for human macrophages [130]. As antagonistic LPS molecules have the potential to limit endotoxin-induced inflammatory symptoms, they are of great interest for the pharmaceutical field, and have been receiving attention for therapeutic applications [3]. Notably, bacteria are able to change the structure and composition of their LPS to better adapt to the environment and as part of their immune evasion mechanisms. For instance, Kawahara and co-workers observed that *Yersinia pestis* can perform a shift from the highly inflammatory hexa-acylated LPS form, produced at 27 °C during its life cycle in the vector flea, to low-to-non-immunogenic tetra-acylated lipid A, which is synthesized at 37 °C, the typical temperature found in warm-blooded hosts such as humans or rodents. As a result, the immune response against the pathogen is weakened, and *Yersinia pestis* can spread throughout the body causing a disease commonly known as plague [41,131,132,133] (Figure 4).

Similar phenomena have been noted for species such as *Helicobacter pylori* and *Porphyromonas gingivalis*, which are both associated with immune evasion and chronic infection [134,135,136]. In summary, the heterogeneity of LPS is an important aspect in regards to immune activation that should not be underestimated. Structural differences, especially regarding the lipid A moiety, can have a significant impact on innate immune binding, and consequently, biological activity.

## 4. LPS Detection: Methods, Challenges, and Future Options

### 4.1. Limitations of Detection Assays

**Figure 5:**
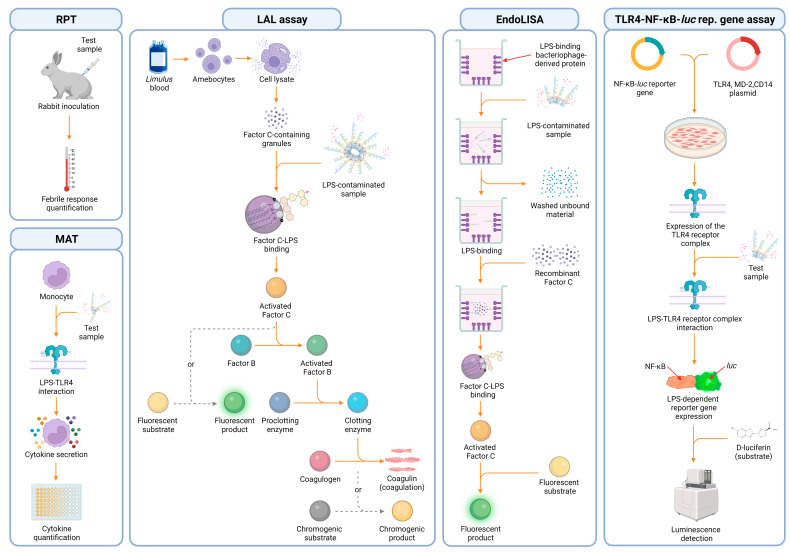
Graphical scheme depicting the underlying mechanisms in different LPS detection methods. From left to right: rabbit pyrogen test (RPT), monocyte activation test (MAT), gel clot limulus amebocyte lysate (LAL) assay and its variants (e.g., chromogenic LAL), EndoLISA, and, ultimately, the TLR4-NF-*κ*B-luciferase (TLR4-NF-*κ*B-luc) reporter gene assay.

Various methods are used to detect endotoxins. Among these, the rabbit pyrogen test (RPT) is the oldest one, approved by the U.S. food and drug administration (FDA). In this in vivo test, rabbits are inoculated with the sample, and the febrile response is used as a positive readout for pyrogen contamination (Figure 5). However, due to its high costs, animal welfare concerns, and low specificity to endotoxin the RPT is being substituted by more efficient, cost-effective methods [9,122]. Additionally, there is a plan to eventually remove this test from the EU pharmacopeia by 2026 [137]. Another animal-based method is the gel-clot limulus amebocyte lysate (LAL) assay, which exploits a naturally evolved defense mechanism of the Atlantic Horseshoe Crab (*Limulus polyphemus*) to detect LPS. The limulus’ blood is, in fact, rich in innate immune cells called amebocytes, which are responsible for defending it against pathogens such as (G-) bacteria [122,138]. These cells are packed with cytoplasmic granules formed by a zymogen called factor C. This protein can be autocatalytically activated by LPS during infection, causing the initiation of a coagulation cascade that culminates in the conversion of coagulogen into coagulin and the formation of immobilizing and inhibiting clots around bacteria, aiming for infection containment (Figure 5) [138,139,140,141,142]. This unique and effective ability was the foundation of the LAL assay. Factor C is thereby extracted from lysed amebocytes and used as an indicator for LPS contamination [142,143,144]. However, this coagulation-based method enables only an imprecise, eye-based quantification of the results. On that account, other machine-based detection methods were developed, such as the turbidimetric and the chromogenic LAL assay (Figure 5) [145,146,147]. Similarly, the *Tachypleus* amebocyte lysate (TAL) assay uses amebocytes extracted from other arthropods species (*Tachypleus gigas* or *Tachypleus tridentatus*). It is also suitable for endotoxin detection and quantification, although its use is mainly limited to Asian countries [148]. One major advantage of the LAL test is that the triggered cascade strongly amplifies the signal, allowing the detection of low endotoxin concentrations down to 0.01 endotoxin unit (EU)/mL. However, the clotting enzyme can also be activated by other substances, such as (1→3)-*β*-d-glucan, leading to false positive results. Additionally, its sensitivity can be reduced by the type and source of LPS being detected, by the sample processing, and by the presence of chelating agents, antibodies, LBP, cationic proteins, surfactants, as well as blood proteins [9,90,144,149,150,151,152,153,154]. Thus, new assays were developed to overcome those limitations, such as the EndoLISA. In this test, similar to a conventional enzyme-linked immunosorbent assay (ELISA), samples are added to plastic wells coated with a bacteriophage-derived receptor protein, which can capture LPS via its core region [153]. LPS is then detected using a recombinantly produced factor C, which then processes a substrate to generate a machine-quantifiable fluorescent signal (Figure 5) [153,155,156].

Despite many advantages, its broad sensitivity (0.05–500 EU/mL), its superior robustness in terms of endotoxin spike recovery under different stress-inducing conditions, its insensitivity to glucan contaminations, and the use of animal-friendly recombinant proteins, EndoLISA has some major drawbacks. It can only detect LPS in liquid samples [157] and it is still affected by the presence of chelating agents similar to a conventional LAL assay [90,153,154]. This development was followed by other ELISA-based assays that require LPS to be adsorbed to plastic wells or to be detected by different capture proteins, such as monoclonal antibodies (mAbs) or the antibiotic polymyxin B (PMB) [9,158,159]. In this context, Appelmelk et al. developed a PMB–horseradish peroxidase conjugate that effectively improved LPS quantification from different sources in direct and sandwich-ELISA formats [160]. Meanwhile, Scott et al. used PMB-functionalized plastic wells to improve endotoxin coating efficiency and develop tests to detect anti-LPS IgG in patients affected by (G-) bacterial infections [161]. This is particularly useful because the limited capacity of amphiphilic LPS to adsorb onto plastic in the presence of proteins is one of the reasons why ELISA-based tests are prone to low specificity and result in inconsistency when investigating complex samples. In fact, protein affinity for plastic surfaces easily outcompetes LPS adsorption, further reducing detection capacity [9]. Additionally, the use of anti-O-antigen mAbs as capture/detection proteins is prone to cross-reactivity, and their production is often laborious and expensive since many LPS variants have not yet been isolated [9,162]. Other alternatives to conventional LPS detection methods are cell-based assays, such as the monocyte activation test (MAT), and two reporter gene assays, known as the TLR4-NF-*κ*B-luciferase reporter gene assay and the commercially available HEK-blue assay (InvivoGen, San Diego, USA) (lower detection limit of 0.1 EU/mL, 0.1 EU/mL, and 0.01 EU/mL, respectively). In the MAT, monocytes are exposed to samples, and the release of IL-1*β* and IL-6 is used as an indication of LPS contamination. In reporter gene assays, cells are transfected with plasmids containing the TLR4 receptor complex and a reporter gene (NF-*κ*B-luciferase or NF-*κ*B-SEAP) instead, which produces a luminescence signal or a colorimetric reaction upon LPS recognition by TLR4 [90,163] (Figure 5). Both the MAT and the luciferase reporter gene assay have been shown to reflect the natural response to LPS to be high throughput, species-specific, and less sensitive to endotoxin masking, with MAT also being capable of detecting contaminations on solid surfaces [90,157]. However, using viable cells is demanding and the results can be affected by the presence of any other immunomodulatory or cytotoxic molecule in the tested substances. Furthermore, non-hexa-acylated lipid A can be less stimulatory, and therefore, shift results towards false negatives, despite high LPS concentrations [122]. As a consequence, and as outlined in the previous chapter, the composition of lipid A’s structure can have an immense impact on the detection. Thus, we can expect that the sensitivity of cell-based assays, which exploit the TLR4 receptor system, can be extremely variable when different LPS variants are under investigation. In addition to this, MAT kits commonly use more complex cellular sources, which are not made solely of pure monocyte samples. This can potentially influence result consistency and needs to be taken into consideration before this method is selected. For example, the PyroDetect Kit (Merck, Darmstadt, Germany) provides whole blood as a monocyte source, MAT Biotec uses PBMCs pooled from four to eight donors (MAT Biotec, Abcoude, Netherlands), while some others, use the Mono Mac 6 cell line (EuroFins, Luxembourg city, Luxembourg), which was derived from a patient affected by acute monocytic leukemia. Thus, identifying a suitable assay for the given sample is critical to ensure product safety and result consistency.

### 4.2. Low Endotoxin Recovery and Endotoxin Potency

Although all detection assays have advantages and disadvantages, there are some overarching issues linked directly to LPS properties, which insert an additional layer of complexity in endotoxin detection and quantification. In quality control procedures, a control standard endotoxin (CSE) [151] is used to build a calibration curve aiming at the correct quantification of endotoxin on a test sample. The calibration curve is expressed in endotoxin units/volume (e.g., EU/mL). The unit EU, however, is rather related to the endotoxin potency than to its mass, and it is directly dependent on both the chemical structure of LPS and the method employed for the quantification [164]. Yet, in research, the amount of LPS used during an experiment is usually expressed in mass/volume (e.g., ng/mL) and not EU/volume, which can potentially lead to incorrect LPS quantification since LPS strains differ in their mass and endotoxin potency [164]. In the literature, in fact, different correlations between EU and mass, even for LPS from the same bacterial strain, are reported. For instance, while both Petsch et al. and Magalhães et al. used LPS from *Escherichia coli* O111:B4 in their publications, Petsch et al. considered 0.1 ng of LPS equal to 1.2 EU, whereas Magalhães et al. considered 0.12 ng of LPS equal to 1 EU [80,91]. In the low contamination range, this difference might not be relevant; however, when higher contaminations are present in diluted test samples, this can lead to a significantly different outcome. Another challenge in LPS quantification was described by Chen and Vinther in 2013: the inability of endotoxin quantification tests to recover more than 50% of a spiked LPS amount on undiluted samples. This phenomenon was called low endotoxin recovery (LER) [151,165]. Unlike test interferences, LER cannot be reversed by dilutions, and it is very important in pharmaceutical hold-time studies, as it was shown to be a time-dependent effect [90,151,166,167]. This means that in a LER solution, the endotoxin potency is not constant throughout, but it is strongly influenced by extrinsic factors that will affect the endotoxin recovery rate. The following factors were already reported to contribute to LER: temperature, pH, salinity, the presence of chelating agents (e.g., sodium citrate and EDTA), surfactants (e.g., Tween-20, Triton X-100, and polysorbate 20 and 80), and cationic proteins (e.g., lysozyme, ribonuclease A, and human IgG). These factors relate to both the sample and the buffers used during sample preparation or analysis [90,91,148,150,151,154,166,168,169]. Those factors can separate LPS from micellar into a less active monomeric form [148,150,167], or even interact directly with LPS, reducing its activity and solubility [122,170]. In an interesting approach, the LER of naturally occurring endotoxin (NOE) and CSE was evaluated by Schwarz et al., who compared the LPS recovery in different buffers known to induce LER effects. The buffers were composed of 10 mM sodium citrate and either 0.05% of a surfactant (Tween-20 or Triton X-100) or 10 mg/mL BSA. The authors demonstrated that although the response of the cell-based assays can be reduced with the presence of LER-inducing buffers, the masked LPS was still biologically active. However, it was not able to induce a response in factor C-based tests (LAL and recombinant factor C) [90]. Therefore, a case-by-case strategy needs to be considered, which involves the best approach to measuring endotoxin contamination, as well as a suitable CSE as a reference. This would enhance the safety standards for medical products and result in consistency in research.

### 4.3. LPS-Binding Molecules

The search for high-affinity binders that target endotoxin has been a popular research field for several decades. It has the potential to open doors for many applications in various disciplines. Among these disciplines, the medical sector can benefit greatly from these advances by using LPS-binding molecules as anti-inflammatory agents [171,172] or tools to remove endotoxin from the blood of sepsis patients [173]. Additionally, binding molecules can be coupled to nanoparticles to be employed as sensing tools [174,175,176]. Choosing the right binding molecule for each application is essential to achieve the best results while assuring safety for the patient as well as consistent outcomes. A decisive factor for this choice is the heterogeneity of LPS since the molecules’ binding affinity differs between bacterial species due to the diversified interdependency of electrostatic and/or amphipathic interactions [177,178]. To compare the binding properties between molecule and target, the dissociation constant K_D_ is broadly used—it allows to classify the strength of interactions. Table 2 summarizes various LPS-binding molecules of different origins, including the bacterial strain and target, as well as K_D_ values, if stated by the authors. LPS-binding molecules can be found in nature, recombinantly produced or even fully synthetic. Native LPS-binding molecules are found in many living organisms, including insects [179,180,181], arthropods [182,183,184], mammals [185,186,187], and more; a significant number of them are part of the immune system and aim at host protection and pathogen clearance. One of the most popular native LPS binders so far is PMB, an antibiotic expressed by the (G+) bacterium *Paenibacillus polymyxa* [158,159]. PMB has been used as a positive control for neutralization and binding affinity studies [188,189,190], and was used as a starting point by Deris et al. in the development of the semi-synthetic probe MIPS-9451 [191]. Subsequently, McInerney et al. used this probe to screen binding affinities against 17 different LPS strains, and hence, acknowledged the importance of LPS heterogeneity. The great potential of these binders became obvious as the binding capabilities towards 14 of these LPS strains were consistent [176].

Several studies aim to create novel LPS-binding molecules by using native proteins as templates. One example of creating such semi-synthetic binders is the modification of peptide fragments to increase their binding affinity towards LPS. Singh and co-workers used trypsin to digest the known binder lactoferrin in order to find novel LPS-binding fragments. Luckily, this treatment exposed a second binding site on lactoferrin that caused an improvement of the K_D_ value by a factor of 1000 [192]. Furthermore, the heterogeneity of LPS is highlighted by the effect of LPS-binding molecules on different bacterial strains. Here, two major indicators are used for the characterization of binders: the minimum bactericidal concentration (MBC) and the minimum inhibitory concentration (MIC). For instance, recombinant g-type lysozyme (rLysG1) from deep-sea hydrothermal vent shrimp was found to bind LPS, disrupting cell wall stability, and finally, leading to cell lysis and death. However, not every (G-) bacterial strain was affected equally by this binder. Some bacteria—e.g., *Escherichia coli* and *Pseudoalteromonas hodoensis*—were lysed already at low concentrations of rLysG1, whereas others—such as different *Vibrio* strains—were not affected at all [193]. The changes that LPS binders have on the morphology of bacteria can be observed using electron microscopy. Blebbing on the outer bacterial membrane is commonly studied using scanning electron microscopes. The cellular blebbing process is an early-stage sign of cell damage [193,194,195]. Furthermore, LPS binders can have strong effects on cell wall permeability, which was shown through transmission electron microscopy images of *Escherichia coli* 25922 upon treatment with the hornet venom component mastoparan-1 (MP-1). Here, morphological changes of the cell wall were observed after 15 min of incubation with MP-1 [196].

**Table 2:** LPS-binding molecules from several species. (*E.* = *Escherichia*, *S.* = *Salmonella*).

Origin	Binding Molecule	LPS Strain (Serotype)	LPS Target	K_D_ Values	Citation
**Microorganisms**					
Bacteriophage	Bacteriophage Sf6 tailspike protein	*Shigella flexneri*	O-antigen	-	[197]
Bacteriophage	Phage P22 tailspike protein	*S. enterica typhimurium*, *S. enteritidis*	O-antigen	-	[198,199]
Virus	SARS-CoV-2 spike protein	*E. coli*	Lipid A	47 nM	[200]
Gram positive bacteria (*Bacillus polymyxa*)	Polymyxin B (PMB)	*E. coli* (O55:B5), *S. minnesota* (Re 595)	Lipid A	Lipid A 5.6 nM, LPS 25.4 nM	[189]
Gram positive bacteria (*Bacillus polymyxa*)	Polymyxin B (PMB)	*E. coli* (K12)	Lipid A	0.71 µM	[201]
Gram positive bacteria (*Bacillus polymyxa*)	Synthetic polymyxin MIPS-9451	*E. coli* (O111:B4, O26:B6), *S. enterica (abortus equi*, *enteritidis*, *minnesota)*, *Serratia marcescens*, *Helicobacter pylori*, *Porphyromonas gingivalis*, *Klebsiella pneumoniae*, *Pseudomonas aeruginosa*, *Proteus mirabilis*, *Proteus vulgaris*, *Campylobacter jejuni*, *Bordetella pertussis*	Lipid A	0.14 µM– 7.2 µM	[176]
**Insects**					
Honey bee	Melittin	*E. coli* (O111:B4), *E. coli* (F583)	Lipid A	0.3 µM	[181]
Hornet	Masroparan-1 (MP-1)	*E. coli* (O55:B5), *E. coli* (O111:B4), *S. minnesota* (Re 595)	Lipid A	Lipid A 456 nM, LPS 484 nM	[196]
Fly	Attacin	*E. coli* (K12)	Lipid A and inner core	-	[202]
Rove beetle (*Paederus*)	Sarcotoxin Pd	*E. coli*, *Klebsiella pneumoniae*	-	-	[180]
Larvae (*Papilio xuthus*)	Papiliocin	*E. coli* (O111:B4)	-	63 nM	[171,179]
Larvae (*Papilio xuthus*)	N- terminal helix of papiliocin (PapN)	-	Phosphate of lipid A	-	[177]
**Arthropods**					
Horseshoe crab	Factor B	*E. coli* (O111:B4) *S. minnesota* R595	-	3.49 nM 10.3 nM	[183,203]
Horseshoe crab	Factor C	*S. minnesota* R595 (Re)	-	0.758 nM	[183,203]
Horseshoe crab	Factor C	*E. coli* (K12)	Lipid A	0.76 nM	[184]
Horseshoe crab	Tachyplesin I (TP I)	-	Phosphate groups and KDO	under 100 μM	[204]
Horseshoe crab (*Tachypleus* *tridentatus*)	Tachypleus antilipopolysaccharide LPS factor (TALF)	*E. coli* (O111:B4)	Lipid A	-	[182]
Shrimp (*Penaeus monodon*)	Shrimp anti-lipopolysaccharide factor (SALF)	-	-	-	[172]
Shrimp (*Rimicaris* sp.)	G-type lysozyme (LysG1)	*E. coli*, *Psedoalteromonas* *hodoensiswas*	Lipid A	-	[193]
Horseshoe crab (*Carcinoscorpius* *rotundicauda*)	Derived from factor C Sushi-1 Sushi-3	*E. coli* (K-12)	Lipid A	Sushi1 0.14 nM Sushi3 0.39 nM	[201,205]
Horseshoe crab (*Achypleus tridentatus*, *Limulus polyphemus)*	LALF_31–52_	*E. coli* (O111:B4)	Lipid A	47.8 µM	[206]
Horseshoe crab	A synthetic cyclic peptide derived from LALF CLP-19	*E. coli* (O111:B4)	Lipid A	8.26 μM	[206]
**Mammals**					
Human	Human lysozyme (HL)	*Klebsiella pneumoniae* O1	O-antigen	0.41 mM	[207]
Human	Interleukin-8	*Aggregatibacter actinomycetemcomitans*	-	1.2–17 μM	[187]
Human	Human β-defensin 114 (DEFB114)	*E. coli* (O111:B4)	-	0.44 µM	[208]
Human	Human β-defensin 126 (DEFB126)	*E. coli* (O111:B4)	-	54.16 nM	[209]
Human	CD14	*E. coli* (O55:B5)	Lipid A	8.7 µM	[185]
Human	CD14	*S. minnesota* (Re595)	Lipid A	29 nM	[210]
Human	MD-2	*E. coli* (O55:B5)	Lipid A	3.2 µM	[185]
Human	TLR4	*E. coli* (O55:B5)	Lipid A	14.1 µM	[185]
Human	LBP	*S.minnesota* (Re595)	Lipid A	3.5 nM	[210]
Human	rLBP	*E. coli* (J5)	Lipid A	1.05 nM	[211]
Human	5I-histidine-rich polypeptide (Histatin 5)	*Porphyromonas gingivalis*	-	1.5 µM	[212]
Human	Lactoferrin	*E. coli* (various serotypes), *Pseudomonas aeruginosa*, *Klebsiella pneumoniae*, *Neisseria meningitides*, *Neisseria gonorrhoeae*, *Haemophilus influenzae*, *Branhamella catarrhalis*, *Shigella flexneri*, *Helicobacter pylori*	Lipid A	2 nM	[213]
Human	BPI_21_	*S. minnesota* (R595)	Lipid A	3.75 nM	[214,215]
Human	rBPI_23_ rBPI_55_ CAP57	*E. coli* (J5) lipid A, *S. minnesota* (Ra), *E. coli* (O113), *S. abortus*	Lipid A	1.7 nM–5.2 nM	[211]
Human	BNEP (derived from BPI)	LPS *E. coli* (O55:B5); Lipid A *S. minnesota* (Re 595)	Lipid A	LPS 25.8 nM Lipid A 11.8 nM	[189]
Human	LL-37 (derived from CAP-18)	LPS *E. coli* (O111:B4)	-	77.5 nM	[216,217]
Human	Derived from high mobility group box 1 (HMGB1) HPep1 HPep6	*E. coli* (O111:B4), *S. minnesota*, *S. typhimurium*	O-Antigen Lipid A	-	[188,218]
Human	Serum amyloid P component (SAP)	*S. minnesota* (R595)	Lipid A	3.9 nM	[214,215]
Bovine, human	Lactoferrin	*E. coli* (O55:B5)	Lipid A	Human 3.6 nM Bovine 4.5 nM Low-affinity binding site: Human 390 nM	[186]
Bovine	Fragment of lactoferrin (LF); Fragment of lactoferrin = lactosmart	*E. coli* (O26:B6) *Pseudomonas aeruginosa*, *Shigella flexneri*	Phosphate group, KDO and lipid A	0.049 nM LPS/ lactosmart, 32 nM LPS/LF	[192]
Bovine	Derived from neutrophil granules BAC7(1–35)	*E. coli* (O111:B4)	Lipid A	-	[219]
Rabbit	Cationic protein 18 (CAP18)	*S. minnesota* (R595)	Lipid A	0.58 nM	[214,215]
Sheep leukocytes	Sheep myeloid antimicrobial peptide-29 (SMAP-29) or (SC5)	-	-	-	[194]
Porcine (pig) leukocytes	Protegrin-1 (PG-1)	*Neisseria meningitidis*	Phosphate head groups and lipid A	-	[220]
**Birds**					
Chicken	Fowlicidin-1	-	-	-	[221]
Chicken	Lysozyme	*S. minnesota* (R595)	Phosphate groups of lipid A	-	[178]
**Amphibians**					
Frog (*Xenopus laevis*)	Magainin 2	*E. coli*, *Acinetobacter calcoaceticus*	-	-	[222]

Besides the interaction with LPS on living bacteria, one major task of binding molecules is meant to be the interaction with free LPS and the subsequent neutralization. This ability was quantified by adding binder and LPS simultaneously to various cell types, such as neutrophils [223], RAW264.7 macrophages [209], and HeLa cells [172]. A decrease in proinflammatory cytokines was observed, including the cytokines TNF-α, IL-1β, and IL-6 both in vitro [206,209,223] and in vivo [206]. Besides influences on cytokine levels, the binding molecule Cecropin-like antimicrobial peptide (SibaCec) showed a dose-dependent reduction in LPS-induced NO and nitrite production in mouse macrophages [224].

In conclusion, LPS binders struggle with LPS heterogeneity. Therefore, it is crucial to target well-conserved parts of the LPS backbone—a promising candidate could be, for instance, the KDO moiety. KDO is part of the inner core region of LPS and is present in the majority of (G-) bacteria [14]. However, it might be a challenge to only target this molecule in the process of screening for binders, since it could be shielded by other surrounding molecules, sterically blocking access for the target site. Another possibility to overcome the restrictions of LPS binders regarding the heterogeneity is by adjusting the experimental design; for instance, the combination of different binders in one assay could be exploited to create a universal approach instead of a universal binder. This might be achieved by immobilizing binders that target various LPS variants on one detection platform.

## 5. Recommendations for a More Efficient Workflow with LPS

The high variability in the molecular structure of LPS and the subsequent change of behavior in different solutions creates a huge challenge in research and development as well as in pharmaceutical sciences. It is, therefore, important to carefully evaluate the exact working conditions and even the type of containers used for reagents. Studies showed that there was a higher LPS recovery in glassware, polystyrene, and PETG [225] compared to other plastics, such as polycarbonate and polypropylene [225,226]. Since LPS is able to bind proteins non-specifically, the selection of an optimal blocking agent is crucial for success in experimental procedures, such as during ELISA or when blocking free surfaces in biosensor assays. Mostly, BSA is used as a standard protein for this purpose; however, it is known that BSA can interact with LPS [93]. Therefore, the selection of alternative proteins may become relevant for the outcome of many types of experiments. In a study by Péterfi et al., goat serum outperformed BSA or casein as a blocking reagent for the detection of different (S) and (R) forms in ELISA [227]. Regarding storage conditions, LPS can safely be stored at −20 °C for several months; however, freeze/thaw cycles must be avoided to assure an intact molecule. Studies showed that repeated freeze/thaw cycles can elicit a conformational alteration in the phosphate-heptose region [228], which can lead to a loss of its biological potency by 25% after every cycle [229]. Nevertheless, LPS that was stored at 7 °C for more than a year showed no loss in endotoxin activity [229]. Additionally, (DR) LPS exhibited a higher sensitivity towards freeze/thaw cycles than wild-type or other less rough LPS types [230]. Comparing the biological activities of different LPS types is a challenge. The quality and purity of the isolates are still a major difficulty. Depending on the LPS structure, the extraction methods differ in their efficiency. Nguyen and coworkers improved the quality of extraction and the biological activity of different LPS types with a novel method called T-sol that includes trizol-like solutions [231]. However, even commercially available LPS extracts still comprise protein contaminations, which can lead to a misinterpretation of experimental results. Thus, it is recommended to quantify the protein content in LPS using a protein quantification method, such as the bicinchoninic acid assay [232]. Along with this, many articles are lacking crucial information about specific LPS type, buffers for LPS preparation, or the conversion factor from ng to EU. Hence, for a better comparison of different studies and to increase the chance to correlate results, this information needs to be reported in every publication in line with the concept of metadata stewardship [233].

## 6. Conclusions

Over the last decades, the interest in understanding morphological features of LPS and their impact on immunological activation, detection, removal, and neutralization has risen immensely, which can be demonstrated by the fact that the term “endotoxin” achieved more than 5000 new hits, and the term “lipopolysaccharide” achieved 7800 hits in PubMed in 2022 [234]. However, the broad heterogeneity of these molecules imposes a multi-layered challenge in comparing different studies. This is of concern, as several cytotoxicity studies use LPS as a positive control for immune response activation, but there is no standardization yet, neither in the handling and storage nor in the type of LPS or the bacterial strain used. Information is often missing in publications, although essential for comparing different assay outcomes. Hitherto, a great knowledge gap exists about LPS heterogeneity and its impact on the studies’ results. In order to advance LPS-related assays, this gap needs to be filled to overcome the present limitations for the design of affordable, portable, simple, and comprehensive LPS detection kits that can be deployed in complex media (including salts, sugars, and particles). Combining various binding molecules that are able to distinguish LPS subtypes in one single assay could revolutionize novel diagnostic tools and widen existing applications in the medical field.

## Figures and Tables

**Table 1:** Structural properties of lipid A variants. The table includes the number and distribution of sugar groups (*) in the form of “a (b + c)”, where a is the total number of sugars, b is the number of groups attached to GlcN II, and c the number attached to GlcN I. Furthermore, the number of phosphate groups (**) is given; groups are distributed symmetrically if not stated otherwise. If no information is given for the agonistic activity (***), the literature did not provide data. For bacteria, most abundant structure is denoted as “major”, others as “minor”.

Bacteria	Sugar Groups *	Phosphate Groups **	Lipid A Acyl Chains	Length of Acyl Chains	Agonistic Activity ***	Citation
*Acinetobacter baumannii*		2	6 (4 + 2) 7 (4 + 3)	12–14	Agonist	[46]
*Actinobacillus actinomycetemcomitans*		2	6 (4 + 2)	14	Agonist	[47]
*Alcaligenes faecalis*		2	6 (3 + 3)	10–14	Weak agonist	[48]
*Aquifex pyrophilus*	2 (1 + 1)	0	5 (3 + 2)	14–18	Weak/No agonist	[23]
*Bacteroides fragilis*		1 (0 + 1)	5 (3 + 2)	15–17	Weak agonist	[49]
*Bacteroides vulgatus*		1 (0 + 1)	5 (3 + 2)	15–17	Weak agonist	[50]
*Bartonella quintana*		2	5 (3 + 2)	12–26	Antagonist	[51]
*Bordetella parapertussis*		2	6 (4 + 2)	10–16	Weak/No agonist	[34]
*Bordetella pertussis*		2	5 (3 + 2)	10–14	Agonist	[34]
*Bradyrhizobium elkanii*	3 (2 + 1)	0	6 (4 + 2)	12–28	Weak agonist/ antagonist	[24]
*Brucella* spp.		1 (1 + 0)	7 (4 + 3)	12–16	-	[30]
*Burkholderia multivorans*	2 (1 + 1)	2	5 (3 + 2)	14–16	Agonist	[52]
*Campylobacter jejuni*		2 (2 + 0)	6 (4 + 2)	14–16	Agonist	[53]
*Chlamydia trachomatis*		2	5 (3 + 2)	14–20	Antagonist	[54]
*Chromobacterium violaceum*		2	6 (3 + 3)	10–12	Antagonist	[55]
*Colwellia hornerae*		2	5 (3 + 2)	9–14	-	[56]
*Colwellia piezophila*		2	5 (3 + 2)	9–14	-	[56]
*Echinicola pacifica*	1 (1 + 0)	1 (0 + 1)	4 (2 + 2)	15–17	Antagonist	[26]
*Echinicola vietnamensis*	1 (1 + 0)	1 (0 + 1)	4 (2 + 2)	15–16	Antagonist	[26]
*Escherichia coli*		2	6 (4 + 2)	12–14	Agonist	[57]
*Escherichia coli* (12 °C)		2	6 (3 + 3)	12–14	-	[40]
*Francisella tularensis*	1 (0 + 1)	1 (0 + 1)	4 (2 + 2)	16–18	No agonist and no antagonist	[58,59]
*Fusobacterium nucleatum*		2	6 (4 + 2)	14–16	Agonist	[60]
*Haemophilus influenzae*		2	6 (4 + 2)	14	Agonist	[61]
*Halobacteroides lacunaris*		2	6 (3 + 3)	10–12	Antagonist	[62]
*Helicobacter pylori*		Minor 2 Major 1 (0 + 1)	Minor 6 (4 + 2) Major 4 (2 + 2)	Minor 12–18 Major 16–18	Major antagonist	[63]
*Klebsiella pneumoniae*		2	6 (4 + 2)	12–14	Agonist	[64]
*Legionella pneumophila*		2	6 (4 + 2)	14–27	No agonist	[27]
*Liberibacter crescens*		1 (0 + 1)	5 (3 + 2)	14–28	-	[65]
*Marinomonas vaga*		1 (0 + 1)	5 (2 + 3)	10–12	Weak agonist	[66]
*Moraxella catarrhalis*		3 (1 + 2)	7 (3 + 4)	10–12	Agonist	[21,67]
*Neisseria meningitidis*		2	6 (3 + 3)	12–14	Agonist	[68]
*Pasteurella multocida* (major)		1 (1 + 0)	5 (4 + 1)	14	-	[35]
*Pasteurella multocida* (minor)	1 (1 + 0)	2	6 (4 + 2)	14	Agonist	[35]
*Porphyromonas gingivalis*		1 (0 + 1)	5 (3 + 2)	15–17	Weak agonist	[69]
*Proteus mirabilis*	1 (1 + 0)	2	7 (4 + 3)	14–16	-	[70]
*Pseudomonas aeruginosa*		2	6 (3 + 3)	10–12	-	[71]
*Psychrobacter cryohalolentis*		2	6 (4 + 2)	8–12	-	[56]
*Psychromonas marina*		2	6 (4 + 2)	12–14	-	[72]
*Ralstonia eutropha*	1 (1 + 0)	2 (1 + 1)	6 (3 + 3)	14	Weak Agonist	[73]
*Ralstonia mannitolilytica*	2 (1 + 1)	2 (1 + 1)	6 (3 + 3)	14–16	Agonist	[73]
*Ralstonia pickettii*	2 (1 + 1)	2 (1 + 1)	5 (3 + 2)	14	No agonist	[73]
*Rhizobium leguminosarum*	1 (1 + 0)	0	5 (3 + 2)	14–28	-	[25]
*Rhodobacter capsulatus*		3 (1 + 2)	5 (3 + 2)	10–14	Antagonist	[20]
*Rhodobacter sphaeroides*		2	5 (3 + 2)	10–14	Antagonist	[33]
*Salmonella minnesota*		2	7 (4 + 3)	12–16	Agonist	[74]
*Salmonella typhimurium*		2	6 (4 + 2)	12–14	Agonist	[75]
*Serratia marcescens*		2	5 (4 + 1)	14	Agonist	[76]
*Sphaerotilus natans*		3 (2 + 1)	6 (3 + 3)	10–14	Agonist	[22]
*Spiribacter salinus*		2	5 (2 + 3)	10–14	-	[77]
*Vibrio cholerae*		2	6 (4 + 2)	12–14	-	[78]
*Vibrio fischeri*		1 (1 + 0)	5 (3 + 2)	12–14	-	[32]
*Yersinia pestis*		2	25–27 °C: 6 (4 + 2) 37 °C: 4 (2 + 2)	12–16 14	Agonist No agonist	[41,79]
*Zunongwangia profunda*		1 (0 + 1)	Minor 4 (2 + 2) Major 5 (3 + 2)	Minor 15–17 Major 15–17	-	[42]

## References

[1] Banerjee S., van der Heijden M.G.A. (2023). Soil Microbiomes and One Health. Nat. Rev. Microbiol..

[2] Schmid A.K., Allers T., DiRuggiero J. (2020). SnapShot: Microbial Extremophiles. Cell.

[3] Di Lorenzo F., Billod J.-M., Martín-Santamaría S., Silipo A., Molinaro A. (2017). Gram-Negative Extremophile Lipopolysaccharides: Promising Source of Inspiration for a New Generation of Endotoxin Antagonists. Eur. J. Org. Chem..

[4] Merino N., Aronson H.S., Bojanova D.P., Feyhl-Buska J., Wong M.L., Zhang S., Giovannelli D. (2019). Living at the Extremes: Extremophiles and the Limits of Life in a Planetary Context. Front. Microbiol..

[5] Rietschel E.T., Kirikae T., Schade F.U., Mamat U., Schmidt G., Loppnow H., Ulmer A.J., Zähringer U., Seydel U., Di Padova F. (1994). Bacterial Endotoxin: Molecular Relationships of Structure to Activity and Function. FASEB J..

[6] Kolmos H.J. (2006). Panum’s Studies on “Putrid Poison” 1856. Dan. Med. Bull..

[7] Pfeiffer R. (1892). Untersuchungen Über Das Choleragift. Z. Für Hyg. Und Infekt..

[8] ICH Guideline Q4B—Annex 14 to Note for Evaluation and Recommendation of Pharmacopoeial Texts for Use in the ICH Regions on Bacterial Endotoxins Tests. https://www.ema.europa.eu/en/documents/scientific-guideline/draft-ich-guideline-q4b-annex-14-note-evaluation-recommendation-pharmacopoeial-texts-use-ich-regions_en.pdf.

[9] Stromberg L.R., Mendez H.M., Mukundan H. (2017). Detection Methods for Lipopolysaccharides: Past and Present. Escherichia coli—Recent Advances on Physiology, Pathogenesis and Biotechnological Applications.

[10] Stenutz R., Weintraub A., Widmalm G. (2006). The Structures of *Escherichia coli* O-Polysaccharide Antigens. FEMS Microbiol. Rev..

[11] Bengoechea J.A., Najdenski H., Skurnik M. (2004). Lipopolysaccharide O Antigen Status of *Yersinia enterocolitica* O:8 Is Essential for Virulence and Absence of O Antigen Affects the Expression of Other *Yersinia* Virulence Factors. Mol. Microbiol..

[12] Schromm A.B., Brandenburg K., Loppnow H., Moran A.P., Koch M.H.J., Rietschel E.T., Seydel U. (2000). Biological Activities of Lipopolysaccharides Are Determined by the Shape of Their Lipid A Portion. Eur. J. Biochem..

[13] King J.D., Kocíncová D., Westman E.L., Lam J.S. (2009). Lipopolysaccharide Biosynthesis in *Pseudomonas aeruginosa*. Innate Immun..

[14] Raetz C.R.H., Whitfield C. (2002). Lipopolysaccharide Endotoxins. Annu. Rev. Biochem..

[15] Liu B., Furevi A., Perepelov A.V., Guo X., Cao H., Wang Q., Reeves P.R., Knirel Y.A., Wang L., Widmalm G. (2020). Structure and Genetics of *Escherichia Coli* O Antigens. FEMS Microbiol. Rev..

[16] Lukáčová M., Barák I., Kazár J. (2008). Role of Structural Variations of Polysaccharide Antigens in the Pathogenicity of Gram-Negative Bacteria. Clin. Microbiol. Infect..

[17] Pupo E., Lindner B., Brade H., Schromm A.B. (2013). Intact Rough- and Smooth-form Lipopolysaccharides from *Escherichia Coli* Separated by Preparative Gel Electrophoresis Exhibit Differential Biologic Activity in Human Macrophages. FEBS J..

[18] Jansson P.-E., Lindberg B., Lindberg A.A., Wollin R. (1981). Structural Studies on the Hexose Region of the Core in Lipopolysaccharides from Enterobacteriaceae. Eur. J. Biochem..

[19] Amor K., Heinrichs D.E., Frirdich E., Ziebell K., Johnson R.P., Whitfield C. (2000). Distribution of Core Oligosaccharide Types in Lipopolysaccharides from *Escherichia coli*. Infect. Immun..

[20] Krauss J.H., Seydel U., Weckesser J., Mayer H. (1989). Structural Analysis of the Nontoxic Lipid A of *Rhodobacter capsulatus* 37b4. Eur. J. Biochem..

[21] Masoud H., Perry M.B., Richards J.C. (1994). Characterization of the Lipopolysaccharide of *Moraxella catarrhalis*. Eur. J. Biochem..

[22] Masoud H., Urbanik-Sypniewska T., Lindner B., Weckesser J., Mayer H. (1991). The Structure of the Lipid A Component of *Sphaerotilus natans*. Arch. Microbiol..

[23] Plötz B.M., Lindner B., Stetter K.O., Holst O. (2000). Characterization of a Novel Lipid A Containing D-Galacturonic Acid That Replaces Phosphate Residues. J. Biol. Chem..

[24] Komaniecka I., Choma A., Lindner B., Holst O. (2010). The Structure of a Novel Neutral Lipid A from the Lipopolysaccharide of *Bradyrhizobium elkanii* Containing Three Mannose Units in the Backbone. Chem. Eur. J..

[25] Bourassa D.V., Kannenberg E.L., Sherrier D.J., Buhr R.J., Carlson R.W. (2017). The Lipopolysaccharide Lipid A Long-Chain Fatty Acid Is Important for *Rhizobium leguminosarum* Growth and Stress Adaptation in Free-Living and Nodule Environments. Mol. Plant-Microbe Interact..

[26] Pither M.D., Mantova G., Scaglione E., Pagliuca C., Colicchio R., Vitiello M., Chernikov O.V., Hua K.-F., Kokoulin M.S., Silipo A. (2021). The Unusual Lipid A Structure and Immunoinhibitory Activity of LPS from Marine Bacteria *Echinicola Pacifica* KMM 6172T and Echinicola Vietnamensis KMM 6221T. Microorganisms.

[27] Zaehringer U., Knirel Y., Lindner B., Sonesson A., Marre R., Rietschel E.T. (1995). The Lipopolysaccaride of *Legionella pneumophila* Serogroup 1 (Strain Philadelphia1): Chemical structure and biological significance. Prog. Clin. Biol. Res..

[28] Gunn J.S. (2001). Bacterial Modification of LPS and Resistance to Antimicrobial Peptides. J. Endotoxin Res..

[29] Raetz C.R.H., Reynolds C.M., Trent M.S., Bishop R.E. (2007). Lipid A Modification Systems in Gram-Negative Bacteria. Annu. Rev. Biochem..

[30] Cardoso P.G., Macedo G.C., Azevedo V., Oliveira S.C. (2006). *Brucella* Spp. Noncanonical LPS: Structure, Biosynthesis, and Interaction with Host Immune System. Microb. Cell Fact..

[31] Onishi H.R., Pelak B.A., Gerckens L.S., Silver L.L., Kahan F.M., Chen M.-H., Patchett A.A., Galloway S.M., Hyland S.A., Anderson M.S. (1996). Antibacterial Agents That Inhibit Lipid A Biosynthesis. Science.

[32] Phillips N.J., Adin D.M., Stabb E.V., McFall-Ngai M.J., Apicella M.A., Gibson B.W. (2011). The Lipid A from *Vibrio fischeri* Lipopolysaccharide. J. Biol. Chem..

[33] Irvine K.L., Gangloff M., Walsh C.M., Spring D.R., Gay N.J., Bryant C.E. (2014). Identification of Key Residues That Confer *Rhodobacter sphaeroides* LPS Activity at Horse TLR4/MD-2. PLoS ONE.

[34] El Hamidi A., Novikov A., Karibian D., Perry M.B., Caroff M. (2009). Structural Characterization of *Bordetella parapertussis* Lipid A. J. Lipid Res..

[35] Tawab A., Akbar N., Hasssan M., Habib F., Ali A., Rahman M., Jabbar A., Rauf W., Iqbal M. (2020). Mass Spectrometric Analysis of Lipid A Obtained from the Lipopolysaccharide of *Pasteurella multocida*. RSC Adv..

[36] Christian A., Zähringer U. (2002). Chemical Structure of Lipid A—The Primary Immunomodulatory Center of Bacterial Lipopolysaccharide. Trends Glycosci. Glycotechnol..

[37] Miura K., Ueno H., Numa Y., Morita S., Nishimoto M. (2021). Effects of Fatty Acid from Deep-Sea Microorganisms on Lipid Bilayer Membrane Fluidity under High Pressure: Comparison of Branched-Chain and Polyunsaturated Fatty Acid. E3S Web Conf..

[38] Carillo S., Pieretti G., Lindner B., Romano I., Nicolaus B., Lanzetta R., Parrilli M., Corsaro M. (2013). The Lipid A from the Haloalkaliphilic Bacterium *Salinivibrio Sharmensis* Strain BAGT. Mar. Drugs.

[39] Di Lorenzo F., Crisafi F., La Cono V., Yakimov M.M., Molinaro A., Silipo A. (2020). The Structure of the Lipid A of Gram-Negative Cold-Adapted Bacteria Isolated from Antarctic Environments. Mar. Drugs.

[40] Carty S.M., Sreekumar K.R., Raetz C.R.H. (1999). Effect of Cold Shock on Lipid A Biosynthesis in *Escherichia coli*. J. Biol. Chem..

[41] Kawahara K., Tsukano H., Watanabe H., Lindner B., Matsuura M. (2002). Modification of the Structure and Activity of Lipid A in *Yersinia pestis* Lipopolysaccharide by Growth Temperature. Infect. Immun..

[42] Pither M.D., Sun M.-L., Speciale I., Silipo A., Zhang Y.-Z., Molinaro A., Di Lorenzo F. (2022). Structural Determination of the Lipid A from the Deep-Sea Bacterium *Zunongwangia profunda* SM-A87: A Small-Scale Approach. Glycoconj. J..

[43] Ernst R.K., Moskowitz S.M., Emerson J.C., Kraig G.M., Adams K.N., Harvey M.D., Ramsey B., Speert D.P., Burns J.L., Miller S.I. (2007). Unique Lipid A Modifications in *Pseudomonas aeruginosa* Isolated from the Airways of Patients with Cystic Fibrosis. J. Infect. Dis..

[44] Schwartzman J.A., Lynch J.B., Flores Ramos S., Zhou L., Apicella M.A., Yew J.Y., Ruby E.G. (2019). Acidic pH Promotes Lipopolysaccharide Modification and Alters Colonization in a Bacteria–Animal Mutualism. Mol. Microbiol..

[45] Gibbons H.S., Kalb S.R., Cotter R.J., Raetz C.R.H. (2005). Role of Mg^2+^ and pH in the Modification of *Salmonella* Lipid A after Endocytosis by Macrophage Tumour Cells. Mol. Microbiol..

[46] Arroyo L.A., Herrera C.M., Fernandez L., Hankins J.V., Trent M.S., Hancock R.E.W. (2011). The PmrCAB Operon Mediates Polymyxin Resistance in *Acinetobacter baumannii* ATCC 17978 and Clinical Isolates through Phosphoethanolamine Modification of Lipid A. Antimicrob. Agents Chemother..

[47] Masoud H., Weintraub S.T., Wang R., Cotter R., Holt S.C. (1991). Investigation of the Structure of Lipid A from *Actinobacillus actinomycetemcomitans* Strain Y4 and Human Clinical Isolate PO 1021-7. Eur. J. Biochem..

[48] Shimoyama A., Di Lorenzo F., Yamaura H., Mizote K., Palmigiano A., Pither M.D., Speciale I., Uto T., Masui S., Sturiale L. (2021). Lipopolysaccharide from Gut-Associated Lymphoid-Tissue-Resident *Alcaligenes faecalis*: Complete Structure Determination and Chemical Synthesis of Its Lipid A. Angew. Chem. Int. Ed..

[49] Weintraub A., Zähringer U., Wollenweber H.-W., Seydel U., Rietschel E.T. (1989). Structural Characterization of the Lipid A Component of *Bacteroides fragilis* Strain NCTC 9343 Lipopolysaccharide. Eur. J. Biochem..

[50] Di Lorenzo F., Pither M.D., Martufi M., Scarinci I., Guzmán-Caldentey J., Łakomiec E., Jachymek W., Bruijns S.C.M., Santamaría S.M., Frick J.-S. (2020). Pairing *Bacteroides vulgatus* LPS Structure with Its Immunomodulatory Effects on Human Cellular Models. ACS Cent. Sci..

[51] Malgorzata-Miller G., Heinbockel L., Brandenburg K., Van Der Meer J.W.M., Netea M.G., Joosten L.A.B. (2016). *Bartonella quintana* Lipopolysaccharide (LPS): Structure and Characteristics of a Potent TLR4 Antagonist for in-Vitro and in-Vivo Applications. Sci. Rep..

[52] Ieranò T., Cescutti P., Leone M.R., Luciani A., Rizzo R., Raia V., Lanzetta R., Parrilli M., Maiuri L., Silipo A. (2010). The Lipid A of *Burkholderia multivorans* C1576 Smooth-Type Lipopolysaccharide and Its pro-Inflammatory Activity in a Cystic Fibrosis Airways Model. Innate Immun..

[53] Cullen T.W., Trent M.S. (2010). A Link between the Assembly of Flagella and Lipooligosaccharide of the Gram-Negative Bacterium *Campylobacter jejuni*. Proc. Natl. Acad. Sci. USA.

[54] Rund S., Lindner B., Brade H., Holst O. (1999). Structural Analysis of the Lipopolysaccharide from *Chlamydia trachomatis* Serotype L2. J. Biol. Chem..

[55] Stewart I., Schluter P.J., Shaw G.R. (2006). Cyanobacterial Lipopolysaccharides and Human Health—A Review. Environ. Health.

[56] Sweet C.R., Watson R.E., Landis C.A., Smith J.P. (2015). Temperature-Dependence of Lipid a Acyl Structure in *Psychrobacter Cryohalolentis* and Arctic Isolates of *Colwellia hornerae* and *Colwellia piezophila*. Mar. Drugs.

[57] Raetz C.R.H., Guan Z., Ingram B.O., Six D.A., Song F., Wang X., Zhao J. (2009). Discovery of New Biosynthetic Pathways: The Lipid A Story. J. Lipid Res..

[58] Phillips N.J., Schilling B., McLendon M.K., Apicella M.A., Gibson B.W. (2004). Novel Modification of Lipid A of *Francisella tularensis*. Infect. Immun..

[59] Barker J.H., Weiss J., Apicella M.A., Nauseef W.M. (2006). Basis for the Failure of *Francisella tularensis* Lipopolysaccharide to Prime Human Polymorphonuclear Leukocytes. Infect. Immun..

[60] Garcia-Vello P., Di Lorenzo F., Lamprinaki D., Notaro A., Speciale I., Molinaro A., Juge N., De Castro C. (2021). Structure of the O-Antigen and the Lipid A from the Lipopolysaccharide of *Fusobacterium nucleatum* ATCC 51191. ChemBioChem.

[61] Helander I.M., Lindner B., Brade H., Altmann K., Lindberg A.A., Rietschel E.T. (1988). Chemical Structure of the Lipopolysaccharide *Haemophilus influenzae* Strain I-69 Rd-/B+. Eur. J. Biochem..

[62] Di Lorenzo F., Palmigiano A., Paciello I., Pallach M., Garozzo D., Bernardini M.-L., La Cono V., Yakimov M.M., Molinaro A., Silipo A. (2017). The Deep-Sea Polyextremophile *Halobacteroides lacunaris* TB21 Rough-Type LPS: Structure and Inhibitory Activity towards Toxic LPS. Mar. Drugs.

[63] Tran A.X., Stead C.M., Trent M.S. (2005). Remodeling of *Helicobacter pylori* Lipopolysaccharide. J. Endotoxin Res..

[64] Li Y., Yun J., Liu L., Li Y., Wang X. (2016). Identification of Two Genes Encoding for the Late Acyltransferases of Lipid A in *Klebsiella pneumoniae*. Curr. Microbiol..

[65] Black I.M., Heiss C., Jain M., Muszyński A., Carlson R.W., Gabriel D.W., Azadi P. (2021). Structure of Lipopolysaccharide from *Liberibacter crescens* Is Low Molecular Weight and Offers Insight into Candidatus Liberibacter Biology. Int. J. Mol. Sci..

[66] Krasikova I.N., Kapustina N.V., Isakov V.V., Dmitrenok A.S., Dmitrenok P.S., Gorshkova N.M., Solov’eva T.F. (2004). Detailed Structure of Lipid A Isolated from Lipopolysaccharide from the Marine Proteobacterium *Marinomonas vaga* ATCC 27119T. Eur. J. Biochem..

[67] Hassan F., Ren D., Zhang W., Merkel T.J., Gu X.-X. (2012). *Moraxella Catarrhalis* Activates Murine Macrophages through Multiple Toll Like Receptors and Has Reduced Clearance in Lungs from TLR4 Mutant Mice. PLoS ONE.

[68] Wenzel C.Q., St. Michael F., Stupak J., Li J., Cox A.D., Richards J.C. (2010). Functional Characterization of Lpt3 and Lpt6, the Inner-Core Lipooligosaccharide Phosphoethanolamine Transferases from *Neisseria meningitidis*. J. Bacteriol..

[69] Hirschfeld M., Weis J.J., Toshchakov V., Salkowski C.A., Cody M.J., Ward D.C., Qureshi N., Michalek S.M., Vogel S.N. (2001). Signaling by Toll-like Receptor 2 and 4 Agonists Results in Differential Gene Expression in Murine Macrophages. Infect. Immun..

[70] Sidorczyk Z., Zähringer U., Rietschel E.T. (1983). Chemical Structure of the Lipid A Component of the Lipopolysaccharide from a *Proteus mirabilis* Re-Mutant. Eur. J. Biochem..

[71] Huang J.X., Azad M.A.K., Yuriev E., Baker M.A., Nation R.L., Li J., Cooper M.A., Velkov T. (2012). Molecular Characterization of Lipopolysaccharide Binding to Human α-1-Acid Glycoprotein. J. Lipids.

[72] Sweet C.R., Alpuche G.M., Landis C.A., Sandman B.C. (2014). Endotoxin Structures in the Psychrophiles *Psychromonas marina* and *Psychrobacter cryohalolentis* Contain Distinctive Acyl Features. Mar. Drugs.

[73] Caroff M., Novikov A. (2020). Lipopolysaccharides: Structure, Function and Bacterial Identification. OCL.

[74] Otten E.G., Werner E., Crespillo-Casado A., Boyle K.B., Dharamdasani V., Pathe C., Santhanam B., Randow F. (2021). Ubiquitylation of Lipopolysaccharide by RNF213 during Bacterial Infection. Nature.

[75] Aldapa-Vega G., Moreno-Eutimio M.A., Berlanga-Taylor A.J., Jiménez-Uribe A.P., Nieto-Velazquez G., López-Ortega O., Mancilla-Herrera I., Cortés-Malagón E.M., Gunn J.S., Isibasi A. (2019). Structural Variants of *Salmonella typhimurium* Lipopolysaccharide Induce Less Dimerization of TLR4/MD-2 and Reduced pro-Inflammatory Cytokine Production in Human Monocytes. Mol. Immunol..

[76] Makimura Y., Asai Y., Sugiyama A., Ogawa T. (2007). Chemical Structure and Immunobiological Activity of Lipid A from *Serratia marcescens* LPS. J. Med. Microbiol..

[77] Barrau C., Di Lorenzo F., Menes R.J., Lanzetta R., Molinaro A., Silipo A. (2018). The Structure of the Lipid a from the Halophilic Bacterium *Spiribacter salinus* M19-40T. Mar. Drugs.

[78] Herrera C.M., Crofts A.A., Henderson J.C., Pingali S.C., Davies B.W., Stephen M. (2014). The *Vibrio cholerae* VprA-VprB Two-Component System Controls Virulence through Endotoxin Modification. MBio.

[79] Chandler C.E., Harberts E.M., Pelletier M.R., Thaipisuttikul I., Jones J.W., Hajjar A.M., Sahl J.W., Goodlett D.R., Pride A.C., Rasko D.A. (2020). Early Evolutionary Loss of the Lipid A Modifying Enzyme PagP Resulting in Innate Immune Evasion in *Yersinia pestis*. Proc. Natl. Acad. Sci. USA.

[80] de Oliveira Magalhães P., Lopes A.M., Mazzola P.G., Rangel-Yagui C., Penna T.C.V., Pessoa A. (2007). Methods of Endotoxin Removal from Biological Preparations: A Review. J. Pharm. Pharm. Sci..

[81] Santos N.C., Silva A.C., Castanho M.A.R.B., Martins-Silva J., Saldanha C. (2003). Evaluation of Lipopolysaccharide Aggregation by Light Scattering Spectroscopy. ChemBioChem.

[82] Gorbet M.B., Sefton M.V. (2005). Endotoxin: The Uninvited Guest. Biomaterials.

[83] Bergstrand A., Svanberg C., Langton M., Nydén M. (2006). Aggregation Behavior and Size of Lipopolysaccharide from *Escherichia coli* O55:B5. Colloids Surf. B Biointerfaces.

[84] Parikh S.J., Chorover J. (2008). ATR-FTIR Study of Lipopolysaccharides at Mineral Surfaces. Colloids Surf. B Biointerfaces.

[85] Yu L., Tan M., Ho B., Ding J.L., Wohland T. (2006). Determination of Critical Micelle Concentrations and Aggregation Numbers by Fluorescence Correlation Spectroscopy: Aggregation of a Lipopolysaccharide. Anal. Chim. Acta.

[86] Ribi E., Anacker R.L., Brown R., Haskins W.T., Malmgren B., Milner K.C., Rudbach J.A. (1966). Reaction of Endotoxin and Surfactants. J. Bacteriol..

[87] Bello G., Eriksson J., Terry A., Edwards K., Lawrence M.J., Barlow D., Harvey R.D. (2015). Characterization of the Aggregates Formed by Various Bacterial Lipopolysaccharides in Solution and upon Interaction with Antimicrobial Peptides. Langmuir.

[88] Hong L., Gontsarik M., Amenitsch H., Salentinig S. (2022). Human Antimicrobial Peptide Triggered Colloidal Transformations in Bacteria Membrane Lipopolysaccharides. Small.

[89] Parikh S.J., Chorover J. (2007). Infrared Spectroscopy Studies of Cation Effects on Lipopolysaccharides in Aqueous Solution. Colloids Surf. B Biointerfaces.

[90] Schwarz H., Gornicec J., Neuper T., Parigiani M.A., Wallner M., Duschl A., Horejs-Hoeck J. (2017). Biological Activity of Masked Endotoxin. Sci. Rep..

[91] Petsch D., Deckwer W.-D., Anspach F.B. (1998). Proteinase K Digestion of Proteins Improves Detection of Bacterial Endotoxins by the Limulus Amebocyte Lysate Assay: Application for Endotoxin Removal from Cationic Proteins. Anal. Biochem..

[92] Kaca W., Roth R.I., Levin J. (1994). Hemoglobin, a Newly Recognized Lipopolysaccharide (LPS)-Binding Protein That Enhances LPS Biological Activity. J. Biol. Chem..

[93] Esparza G.A., Teghanemt A., Zhang D., Gioannini T.L., Weiss J.P. (2012). Endotoxin·albumin Complexes Transfer Endotoxin Monomers to MD-2 Resulting in Activation of TLR4. Innate Immun..

[94] Bello G., Bodin A., Lawrence M.J., Barlow D., Mason A.J., Barker R.D., Harvey R.D. (2016). The Influence of Rough Lipopolysaccharide Structure on Molecular Interactions with Mammalian Antimicrobial Peptides. BBA—Biomembr..

[95] Poltorak A., He X., Smirnova I., Liu M., Van Huffel C., Du X., Birdwell D., Alejos E., Silva M., Galanos C. (1998). Defective LPS Signaling in C3H/HeJ and C57BL/10ScCr Mice: Mutations in TLR4 Gene. Science.

[96] Hoshino K., Takeuchi O., Kawai T., Sanjo H., Ogawa T., Takeda Y., Takeda K., Akira S. (1999). Cutting Edge: Toll-Like Receptor 4 (TLR4)-Deficient Mice Are Hyporesponsive to Lipopolysaccharide: Evidence for TLR4 as the Lps Gene Product. J. Immunol..

[97] Kawasaki T., Kawai T. (2014). Toll-Like Receptor Signaling Pathways. Front. Immunol..

[98] Noh J., Yoon S.R., Kim T., Choi I., Jung H. (2020). Toll-Like Receptors in Natural Killer Cells and Their Application for Immunotherapy. J. Immunol. Res..

[99] Otte J.-M., Rosenberg I.M., Podolsky D.K. (2003). Intestinal Myofibroblasts in Innate Immune Responses of the Intestine. Gastroenterology.

[100] Nagai Y., Akashi S., Nagafuku M., Ogata M., Iwakura Y., Akira S., Kitamura T., Kosugi A., Kimoto M., Miyake K. (2002). Essential Role of MD-2 in LPS Responsiveness and TLR4 Distribution. Nat. Immunol..

[101] Park B.S., Song D.H., Kim H.M., Choi B.-S., Lee H., Lee J.-O. (2009). The Structural Basis of Lipopolysaccharide Recognition by the TLR4–MD-2 Complex. Nature.

[102] Michelini S., Barbero F., Prinelli A., Steiner P., Weiss R., Verwanger T., Andosch A., Lütz-Meindl U., Puntes V.F., Drobne D. (2021). Gold Nanoparticles (AuNPs) Impair LPS-Driven Immune Responses by Promoting a Tolerogenic-like Dendritic Cell Phenotype with Altered Endosomal Structures. Nanoscale.

[103] Scott A.J., Oyler B.L., Goodlett D.R., Ernst R.K. (2017). Lipid A Structural Modifications in Extreme Conditions and Identification of Unique Modifying Enzymes to Define the Toll-like Receptor 4 Structure-Activity Relationship. BBA—Mol. Cell Biol. Lipids.

[104] Kagan J.C., Su T., Horng T., Chow A., Akira S., Medzhitov R. (2008). TRAM Couples Endocytosis of Toll-like Receptor 4 to the Induction of Interferon-β. Nat. Immunol..

[105] Palsson-McDermott E.M., O’Neill L.A.J. (2004). Signal Transduction by the Lipopolysaccharide Receptor, Toll-like Receptor-4. Immunology.

[106] Vanaja S.K., Russo A.J., Behl B., Banerjee I., Yankova M., Deshmukh S.D., Rathinam V.A.K. (2016). Bacterial Outer Membrane Vesicles Mediate Cytosolic Localization of LPS and Caspase-11 Activation. Cell.

[107] Barker J.H., Weiss J.P. (2019). Detecting Lipopolysaccharide in the Cytosol of Mammalian Cells: Lessons from MD-2/TLR4. J. Leukoc. Biol..

[108] Jan A.T. (2017). Outer Membrane Vesicles (OMVs) of Gram-Negative Bacteria: A Perspective Update. Front. Microbiol..

[109] Zamyatina A., Heine H. (2020). Lipopolysaccharide Recognition in the Crossroads of TLR4 and Caspase-4/11 Mediated Inflammatory Pathways. Front. Immunol..

[110] Deng M., Tang Y., Li W., Wang X., Zhang R., Zhang X., Zhao X., Liu J., Tang C., Liu Z. (2018). The Endotoxin Delivery Protein HMGB1 Mediates Caspase-11-Dependent Lethality in Sepsis. Immunity.

[111] Yang H., Wang H., Andersson U. (2020). Targeting Inflammation Driven by HMGB1. Front. Immunol..

[112] Matikainen S., Nyman T.A., Cypryk W. (2020). Function and Regulation of Noncanonical Caspase-4/5/11 Inflammasome. J. Immunol..

[113] Meseguer V., Alpizar Y.A., Luis E., Tajada S., Denlinger B., Fajardo O., Manenschijn J.-A., Fernández-Peña C., Talavera A., Kichko T. (2014). TRPA1 Channels Mediate Acute Neurogenic Inflammation and Pain Produced by Bacterial Endotoxins. Nat. Commun..

[114] Mazgaeen L., Gurung P. (2020). Recent Advances in Lipopolysaccharide Recognition Systems. Int. J. Mol. Sci..

[115] Becker M.N., Diamond G., Verghese M.W., Randell S.H. (2000). CD14-Dependent Lipopolysaccharide-Induced β-Defensin-2 Expression in Human Tracheobronchial Epithelium. J. Biol. Chem..

[116] Håversen L., Ohlsson B.G., Hahn-Zoric M., Hanson L.Å., Mattsby-Baltzer I. (2002). Lactoferrin Down-Regulates the LPS-Induced Cytokine Production in Monocytic Cells via NF-*κ*B. Cell. Immunol..

[117] Hardie E.M., Kruse-Elliott K. (1990). Endotoxic Shock. J. Vet. Intern. Med..

[118] Duerr C.U., Zenk S.F., Chassin C., Pott J., Gütle D., Hensel M., Hornef M.W. (2009). O-Antigen Delays Lipopolysaccharide Recognition and Impairs Antibacterial Host Defense in Murine Intestinal Epithelial Cells. PLOS Pathog..

[119] Rapicavoli J.N., Blanco-Ulate B., Muszyński A., Figueroa-Balderas R., Morales-Cruz A., Azadi P., Dobruchowska J.M., Castro C., Cantu D., Roper M.C. (2018). Lipopolysaccharide O-Antigen Delays Plant Innate Immune Recognition of *Xylella fastidiosa*. Nat. Commun..

[120] Hotchkiss R.S., Moldawer L.L., Opal S.M., Reinhart K., Turnbull I.R., Vincent J. (2016). Sepsis and Septic Shock. Nat. Rev. Dis. Prim..

[121] Mann P.B., Wolfe D., Latz E., Golenbock D., Preston A., Harvill E.T. (2005). Comparative Toll-Like Receptor 4-Mediated Innate Host Defense to *Bordetella* Infection. Infect. Immun..

[122] Gorman A., Golovanov A.P. (2022). Lipopolysaccharide Structure and the Phenomenon of Low Endotoxin Recovery. Eur. J. Pharm. Biopharm..

[123] Gangloff S.C., Hijiya N., Haziot A., Goyert S.M. (1999). Lipopolysaccharide Structure Influences the Macrophage Response via CD14-Independent and CD14-Dependent Pathways. Clin. Infect. Dis..

[124] Meng J., Lien E., Golenbock D.T. (2010). MD-2-Mediated Ionic Interactions between Lipid A and TLR4 Are Essential for Receptor Activation. J. Biol. Chem..

[125] Lagrange B., Benaoudia S., Wallet P., Magnotti F., Provost A., Michal F., Martin A., Di Lorenzo F., Py B.F., Molinaro A. (2018). Human Caspase-4 Detects Tetra-Acylated LPS and Cytosolic *Francisella* and Functions Differently from Murine Caspase-11. Nat. Commun..

[126] Arenas J., Pupo E., Phielix C., David D., Zariri A., Zamyatina A., Tommassen J., van der Ley P. (2020). Shortening the Lipid A Acyl Chains of *Bordetella Pertussis* Enables Depletion of Lipopolysaccharide Endotoxic Activity. Vaccines.

[127] Ingalls R.R., Rice P.A., Qureshi N., Takayama K., Lin K., Golenbock D.T. (1995). The Inflammatory Cytokine Response to *Chlamydia Trachomatis* Infection Is Endotoxin Mediated. Infect. Immun..

[128] Jarvis B.W., Lichenstein H., Qureshi N. (1997). Diphosphoryl Lipid A from *Rhodobacter sphaeroides* Inhibits Complexes That Form in Vitro between Lipopolysaccharide (LPS)-Binding Protein, Soluble CD14, and Spectrally Pure LPS. Infect. Immun..

[129] Lembo-Fazio L., Billod J.-M., Di Lorenzo F., Paciello I., Pallach M., Vaz-Francisco S., Holgado A., Beyaert R., Fresno M., Shimoyama A. (2018). *Bradyrhizobium* Lipid A: Immunological Properties and Molecular Basis of Its Binding to the Myeloid Differentiation Protein-2/Toll-Like Receptor 4 Complex. Front. Immunol..

[130] Muroi M., Tanamoto K. (2006). Structural Regions of MD-2 That Determine the Agonist-Antagonist Activity of Lipid IVa. J. Biol. Chem..

[131] Matsuura M., Takahashi H., Watanabe H., Saito S., Kawahara K. (2010). Immunomodulatory Effects of *Yersinia Pestis* Lipopolysaccharides on Human Macrophages. Clin. Vaccine Immunol..

[132] Rebeil R., Ernst R.K., Gowen B.B., Miller S.I., Hinnebusch B.J. (2004). Variation in Lipid A Structure in the Pathogenic *Yersiniae*. Mol. Microbiol..

[133] Maeshima N., Fernandez R.C. (2013). Recognition of Lipid A Variants by the TLR4-MD-2 Receptor Complex. Front. Cell. Infect. Microbiol..

[134] Mandell L., Moran A.P., Cocchiarella A., Houghton J., Taylor N., Fox J.G., Wang T.C., Kurt-Jones E.A. (2004). Intact Gram-Negative *Helicobacter pylori*, *Helicobacter felis*, and *Helicobacter hepaticus* Bacteria Activate Innate Immunity via Toll-Like Receptor 2 but Not Toll-Like Receptor 4. Infect. Immun..

[135] Netea M.G., Van Deuren M., Kullberg B.J., Cavaillon J.M., Van Der Meer J.W.M. (2002). Does the Shape of Lipid A Determine the Interaction of LPS with Toll-like Receptors?. Trends Immunol..

[136] Rangarajan M., Aduse-Opoku J., Paramonov N., Hashim A., Bostanci N., Fraser O.P., Tarelli E., Curtis M.A. (2008). Identification of a Second Lipopolysaccharide in *Porphyromonas Gingivalis* W50. J. Bacteriol..

[137] EDQM European Pharmacopoeia to Put an End to the Rabbit Pyrogen Test. https://www.edqm.eu/en/-/european-pharmacopoeia-to-put-an-end-to-the-rabbit-pyrogen-test.

[138] Kawabata S., Shibata T. (2022). New Insights into the Hemolymph Coagulation Cascade of Horseshoe Crabs Initiated by Autocatalytic Activation of a Lipopolysaccharide-Sensitive Zymogen. Dev. Comp. Immunol..

[139] Koshiba T., Hashii T., Kawabata S. (2007). A Structural Perspective on the Interaction between Lipopolysaccharide and Factor C, a Receptor Involved in Recognition of Gram-Negative Bacteria. J. Biol. Chem..

[140] Levin J., Williams K.L. (2019). Discovery and Early Development of the Limulus Test.

[141] Wang C., Nelson T., Chen D., Ellis J.C., Abbott N.L. (2019). Understanding Lipopolysaccharide Aggregation and Its Influence on Activation of Factor C. J. Colloid Interface Sci..

[142] Iwanaga S., Miyata T., Tokunaga F., Muta T. (1992). Molecular Mechanism of Hemolymph Clotting System in *Limulus*. Thromb. Res..

[143] Iwanaga S., Kawabata S.-I., Muta T. (1998). New Types of Clotting Factors and Defense Molecules Found in Horseshoe Crab Hemolymph: Their Structures and Functions. J. Biochem..

[144] Ketchum P.A., Novitsky T.J. (2000). Assay of Endotoxin by Limulus Amebocyte Lysate. Septic Shock Methods and Protocols.

[145] Novitsky T.J. (1994). *Limulus* Amebocyte Lysate (LAL) Detection of Endotoxin in Human Blood. J. Endotoxin Res..

[146] Sandle T. (2016). Endotoxin and Pyrogen Testing. Pharmaceutical Microbiology.

[147] Iwanaga S., Morita T., Harada T., Nakamura S., Niwa M., Takada K., Kimura T., Sakakibara S. (1978). Chromogenic Substrates for Horseshoe Crab Clotting Enzyme. Pathophysiol. Haemost. Thromb..

[148] Cao Y., Zhang Y., Qiu F. (2021). Low Endotoxin Recovery and Its Impact on Endotoxin Detection. Biopolymers.

[149] Roslansky P.F., Novitsky T.J. (1991). Sensitivity of Limulus Amebocyte Lysate (LAL) to LAL-Reactive Glucans. J. Clin. Microbiol..

[150] Reich J., Lang P., Grallert H., Motschmann H. (2016). Masking of Endotoxin in Surfactant Samples: Effects on Limulus-Based Detection Systems. Biologicals.

[151] Reich J., Weyer F.A., Tamura H., Nagaoka I., Motschmann H. (2019). Low Endotoxin Recovery—Masking of Naturally Occuring Endotoxin. Int. J. Mol. Sci..

[152] Hurley J.C. (1995). Endotoxemia: Methods of Detection and Clinical Correlates. Clin. Microbiol. Rev..

[153] Grallert H., Leopoldseder S., Schuett M., Kurze P., Buchberger B. (2011). EndoLISA^®^: A Novel and Reliable Method for Endotoxin Detection. Nat. Methods.

[154] Bu R., Deng X., Cao Y., Jin J., Mai B., Meng K., Liu X., Chi J.C., Zhang Y., Qiu F. (2021). Effect of Different Sample Treatment Methods on Low Endotoxin Recovery Phenomenon. J. Microbiol. Methods.

[155] Maloney T., Phelan R., Simmons N. (2018). Saving the Horseshoe Crab: A Synthetic Alternative to Horseshoe Crab Blood for Endotoxin Detection. PLoS Biol..

[156] Loverock B., Simon B., Burgenson A., Baines A., Lonza W. (2010). A Recombinant Factor C Procedure for the Detection of Gram-Negative Bacterial Endotoxin. United States Pharmacop. Conv.-Pharmacop. Forum.

[157] Stang K., Fennrich S., Krajewski S., Stoppelkamp S., Burgener I.A., Wendel H.-P., Post M. (2014). Highly Sensitive Pyrogen Detection on Medical Devices by the Monocyte Activation Test. J. Mater. Sci. Mater. Med..

[158] Morrison D.C., Jacobs D.M. (1976). Binding of Polymyxin B to the Lipid A Portion of Bacterial Lipopolysaccharides. Immunochemistry.

[159] Goode A., Yeh V., Bonev B.B. (2021). Interactions of Polymyxin B with Lipopolysaccharide-Containing Membranes. Faraday Discuss..

[160] Appelmelk B.J., Su D., Verweij-van Vught A.M.J.J., Thijs B.G., MacLaren D.M. (1992). Polymyxin B-Horseradish Peroxidase Conjugates as Tools in Endotoxin Research. Anal. Biochem..

[161] Scott B.B., Barclay G.R. (1987). Endotoxin-Polymyxin Complexes in an Improved Enzyme-Linked Immunosorbent Assay for IgG Antibodies in Blood Donor Sera to Gram-Negative Endotoxin Core Glycolipids. Vox Sang..

[162] Trautmann M., Held T.K., Susa M., Karajan M.A., Wulf A., Cross A.S., Marre R. (1998). Bacterial Lipopolysaccharide (LPS)-Specific Antibodies in Commercial Human Immunoglobulin Preparations: Superior Antibody Content of an IgM- Enriched Product. Clin. Exp. Immunol..

[163] Borton L.K., Coleman K.P. (2018). Material-Mediated Pyrogens in Medical Devices: Applicability of the in Vitro Monocyte Activation Test. ALTEX.

[164] Dawson M.E. (1993). Endotoxin Standards and CSE Potency. LAL Updat..

[165] Chen J., Vinther A. (2013). Low Endotoxin Recovery in Common Biologics Products, PDA Annual Meeting.

[166] Tsuchiya M. (2014). Possible Mechanism of Low Endotoxin Recovery. Am. Pharm. Rev..

[167] Tsuchiya M. (2019). Sample Treatments That Solve Low Endotoxin Recovery Issues. PDA J. Pharm. Sci. Technol..

[168] Wespel M., Geiss M., Nägele M., Combé S., Reich J., Studts J., Stolzenberger J. (2022). The Impact of Endotoxin Masking on the Removal of Endotoxin during Manufacturing of a Biopharmaceutical Drug Product. J. Chromatogr. A.

[169] Correa W., Brandenburg K., Zähringer U., Ravuri K., Khan T., Von Wintzingerode F. (2017). Biophysical Analysis of Lipopolysaccharide Formulations for an Understanding of the Low Endotoxin Recovery (LER) Phenomenon. Int. J. Mol. Sci..

[170] Rudbach J.A., Johnson A.G. (1964). Restoration of Endotoxin Activity Following Alteration by Plasma. Nature.

[171] Kim J.-K., Lee E., Shin S., Jeong K., Lee J.-Y., Bae S.-Y., Kim S.-H., Lee J., Kim S.R., Lee D.G. (2011). Structure and Function of Papiliocin with Antimicrobial and Anti-Inflammatory Activities Isolated from the Swallowtail Butterfly, *Papilio xuthus*. J. Biol. Chem..

[172] Lin M.-C., Pan C.-Y., Hui C.-F., Chen J.-Y., Wu J.-L. (2013). Shrimp Anti-Lipopolysaccharide Factor (SALF), an Antimicrobial Peptide, Inhibits Proinflammatory Cytokine Expressions through the MAPK and NF-*κ*B Pathways in LPS-Induced HeLa Cells. Peptides.

[173] Tani T., Shoji H., Guadagni G., Perego A. (2010). Extracorporeal Removal of Endotoxin: The Polymyxin B-Immobilized Fiber Cartridge. Contributions to Nephrology.

[174] Zuo M.Y., Chen L.J., Jiang H., Tan L., Luo Z.F., Wang Y.M. (2014). Detecting Endotoxin with a Flow Cytometry-Based Magnetic Aptasensor. Anal. Biochem..

[175] Thakur M., Dan A. (2021). Poly- l -Lysine-Functionalized Green-Light-Emitting Carbon Dots as a Fluorescence Turn-on Sensor for Ultrasensitive Detection of Endotoxin. ACS Appl. Bio Mater..

[176] McInerney M.P., Roberts K.D., Thompson P.E., Li J., Nation R.L., Velkov T., Nicolazzo J.A. (2016). Quantitation of Polymyxin–Lipopolysaccharide Interactions Using an Image-Based Fluorescent Probe. J. Pharm. Sci..

[177] Durai P., Lee Y., Kim J., Jeon D., Kim Y. (2018). Biophysical Studies Reveal Key Interactions between Papiliocin-Derived PapN and Lipopolysaccharide in Gram-Negative Bacteria. J. Microbiol. Biotechnol..

[178] Brandenburg K., Koch M.H.J., Seydel U. (1998). Biophysical Characterisation of Lysozyme Binding to LPS Re and Lipid A. Eur. J. Biochem..

[179] Krishnan M., Choi J., Jang A., Choi S., Yeon J., Jang M., Lee Y., Son K., Shin S.Y., Jeong M.S. (2022). Molecular Mechanism Underlying the TLR4 Antagonistic and Antiseptic Activities of Papiliocin, an Insect Innate Immune Response Molecule. Proc. Natl. Acad. Sci. USA.

[180] Memarpoor-Yazdi M., Zare-Zardini H., Asoodeh A. (2013). A Novel Antimicrobial Peptide Derived from the Insect *Paederus dermatitis*. Int. J. Pept. Res. Ther..

[181] Bhunia A., Domadia P.N., Bhattacharjya S. (2007). Structural and Thermodynamic Analyses of the Interaction between Melittin and Lipopolysaccharide. BBA—Biomembr..

[182] Kloczewiak M., Black K.M., Loiselle P., Cavaillon J.-M., Wainwright N., Warren H.S. (1994). Synthetic Peptides That Mimic The Binding Site Of Horseshoe Crab Antilipopolysaccharide Factor. J. Infect. Dis..

[183] Kobayashi Y., Takahashi T., Shibata T., Ikeda S., Koshiba T., Mizumura H., Oda T., Kawabata S. (2015). Factor B Is the Second Lipopolysaccharide-Binding Protease Zymogen in the Horseshoe Crab Coagulation Cascade. J. Biol. Chem..

[184] Ariki S., Koori K., Osaki T., Motoyama K., Inamori K., Kawabata S. (2004). A Serine Protease Zymogen Functions as a Pattern-Recognition Receptor for Lipopolysaccharides. Proc. Natl. Acad. Sci. USA.

[185] Shin H.J., Lee H., Park J.D., Hyun H.C., Sohn H.O., Lee D.W., Kim Y.S. (2007). Kinetics of Binding of LPS to Recombinant CD14, TLR4, and MD-2 Proteins. Mol. Cells.

[186] Elass-Rochard E., Roseanu A., Legrand D., Trif M., Salmon V., Motas C., Montreuil J., Spik G. (1995). Lactoferrin-Lipopolysaccharide Interaction: Involvement of the 28-34 Loop Region of Human Lactoferrin in the High-Affinity Binding to *Escherichia Coli* 055B5 Lipopolysaccharide. Biochem. J..

[187] Ahlstrand T., Kovesjoki L., Maula T., Oscarsson J., Ihalin R. (2018). *Aggregatibacter Actinomycetemcomitans* LPS Binds Human Interleukin-8. J. Oral Microbiol..

[188] Youn J.H., Oh Y.J., Kim E.S., Choi J.E., Shin J.-S. (2008). High Mobility Group Box 1 Protein Binding to Lipopolysaccharide Facilitates Transfer of Lipopolysaccharide to CD14 and Enhances Lipopolysaccharide-Mediated TNF-α Production in Human Monocytes. J. Immunol..

[189] Jiang Z., Hong Z., Guo W., Xiaoyun G., Gengfa L., Yongning L., Guangxia X. (2004). A Synthetic Peptide Derived from Bactericidal/Permeability-Increasing Protein Neutralizes Endotoxin in Vitro and in Vivo. Int. Immunopharmacol..

[190] Cirioni O., Giacometti A., Ghiselli R., Bergnach C., Orlando F., Silvestri C., Mocchegiani F., Licci A., Skerlavaj B., Rocchi M. (2006). LL-37 Protects Rats against Lethal Sepsis Caused by Gram-Negative Bacteria. Antimicrob. Agents Chemother..

[191] Deris Z.Z., Swarbrick J.D., Roberts K.D., Azad M.A.K., Akter J., Horne A.S., Nation R.L., Rogers K.L., Thompson P.E., Velkov T. (2014). Probing the Penetration of Antimicrobial Polymyxin Lipopeptides into Gram-Negative Bacteria. Bioconjug. Chem..

[192] Singh J., Vijayan V., Ahmedi S., Pant P., Manzoor N., Singh T.P., Sharma P., Sharma S. (2021). Lactosmart: A Novel Therapeutic Molecule for Antimicrobial Defense. Front. Microbiol..

[193] Luo J.-C., Zhang J., Sun L. (2021). A G-Type Lysozyme from Deep-Sea Hydrothermal Vent Shrimp Kills Selectively Gram-Negative Bacteria. Molecules.

[194] Skerlavaj B., Benincasa M., Risso A., Zanetti M., Gennaro R. (1999). SMAP-29: A Potent Antibacterial and Antifungal Peptide from Sheep Leukocytes. FEBS Lett..

[195] Richard G., Roger MacKenzie C., Henry K.A., Vinogradov E., Christopher Hall J., Hussack G. (2020). Antibody Binding to the O-Specific Antigen of *Pseudomonas Aeruginosa* O6 Inhibits Cell Growth. Antimicrob. Agents Chemother..

[196] Yibin G., Jiang Z., Hong Z., Gengfa L., Liangxi W., Guo W., Yongling L. (2005). A Synthesized Cationic Tetradecapeptide from Hornet Venom Kills Bacteria and Neutralizes Lipopolysaccharide in Vivo and in Vitro. Biochem. Pharmacol..

[197] Kunstmann S., Engström O., Wehle M., Widmalm G., Santer M., Barbirz S. (2020). Increasing the Affinity of an O-Antigen Polysaccharide Binding Site in *Shigella flexneri* Bacteriophage Sf6 Tailspike Protein. Chem.—A Eur. J..

[198] Baxa U., Cooper A., Weintraub A., Pfeil W., Seckler R. (2001). Enthalpic Barriers to the Hydrophobic Binding of Oligosaccharides to Phage P22 Tailspike Protein. Biochemistry.

[199] Andres D., Baxa U., Hanke C., Seckler R., Barbirz S. (2010). Carbohydrate Binding of *Salmonella* Phage P22 Tailspike Protein and Its Role during Host Cell Infection. Biochem. Soc. Trans..

[200] Petruk G., Puthia M., Petrlova J., Samsudin F., Strömdahl A.C., Cerps S., Uller L., Kjellström S., Bond P.J., Schmidtchen A. (2020). SARS-CoV-2 Spike Protein Binds to Bacterial Lipopolysaccharide and Boosts Proinflammatory Activity. J. Mol. Cell Biol..

[201] Tan N.S., Ng M.L.P., Yau Y.H., Chong P.K.W., Ho B., Ding J.L. (2000). Definition of Endotoxin Binding Sites in Horseshoe Crab Factor C Recombinant Sushi Proteins and Neutralization of Endotoxin by Sushi Peptides. FASEB J..

[202] Carlsson A., Nyström T., de Cock H., Bennich H. (1998). Attacin—An Insect Immune Protein—Binds LPS and Triggers the Specific Inhibition of Bacterial Outer-Membrane Protein Synthesis. Microbiology.

[203] Kobayashi Y., Shiga T., Shibata T., Sako M., Maenaka K., Koshiba T., Mizumura H., Oda T., Kawabata S. (2014). The N-Terminal Arg Residue Is Essential for Autocatalytic Activation of a Lipopolysaccharide-Responsive Protease Zymogen. J. Biol. Chem..

[204] Kushibiki T., Kamiya M., Aizawa T., Kumaki Y., Kikukawa T., Mizuguchi M., Demura M., Kawabata S., Kawano K. (2014). Interaction between Tachyplesin I, an Antimicrobial Peptide Derived from Horseshoe Crab, and Lipopolysaccharide. BBA—Proteins Proteom..

[205] Li P., Wohland T., Ho B., Jeak L.D. (2004). Perturbation of Lipopolysaccharide (LPS) Micelles by Sushi 3 (S3) Antimicrobial Peptide: The Importance of an Intermolecular Disulfide Bond in S3 Dimer for Binding, Disruption, and Neutralization of LPS. J. Biol. Chem..

[206] Ren J.-D., Gu J.-S., Gao H.-F., Xia P.-Y., Xiao G.-X. (2008). A Synthetic Cyclic Peptide Derived from Limulus Anti-Lipopolysaccharide Factor Neutralizes Endotoxin in Vitro and in Vivo. Int. Immunopharmacol..

[207] Zhang R., Wu L., Eckert T., Burg-Roderfeld M., Rojas-Macias M.A., Lütteke T., Krylov V.B., Argunov D.A., Datta A., Markart P. (2017). Lysozyme’s Lectin-like Characteristics Facilitates Its Immune Defense Function. Q. Rev. Biophys..

[208] Yu H., Dong J., Gu Y., Liu H., Xin A., Shi H., Sun F., Zhang Y., Lin D., Diao H. (2013). The Novel Human β-Defensin 114 Regulates Lipopolysaccharide (LPS)-Mediated Inflammation and Protects Sperm from Motility Loss. J. Biol. Chem..

[209] Liu H., Yu H., Gu Y., Xin A., Zhang Y., Diao H., Lin D. (2013). Human Beta-Defensin DEFB126 Is Capable of Inhibiting LPS-Mediated Inflammation. Appl. Microbiol. Biotechnol..

[210] Tobias P.S., Soldau K., Gegner J.A., Mintz D., Ulevitch R.J. (1995). Lipopolysaccharide Binding Protein-Mediated Complexation of Lipopolysaccharide with Soluble CD14. J. Biol. Chem..

[211] Gazzano-Santoro H., Parent J.B., Grinna L., Horwitz A., Parsons T., Theofan G., Elsbach P., Weiss J., Conlon P.J. (1992). High-Affinity Binding of the Bactericidal/Permeability-Increasing Protein and a Recombinant Amino-Terminal Fragment to the Lipid A Region of Lipopolysaccharide. Infect. Immun..

[212] Murakami Y. (1991). Binding of a Histidine-Rich Peptide to *Porphyromonas gingivalis*. FEMS Microbiol. Lett..

[213] Appelmelk B.J., An Y.Q., Geerts M., Thijs B.G., de Boer H.A., MacLaren D.M., de Graaff J., Nuijens J.H. (1994). Lactoferrin Is a Lipid A-Binding Protein. Infect. Immun..

[214] De Haas C.J.C., Haas P.J., Van Kessel K.P.M., Van Strijp J.A.G. (1998). Affinities of Different Proteins and Peptides for Lipopolysaccharide as Determined by Biosensor Technology. Biochem. Biophys. Res. Commun..

[215] de Haas C.J.C., van der Tol M.E., Van Kessel K.P.M., Verhoef J., Van Strijp J.A.G. (1998). A Synthetic Lipopolysaccharide-Binding Peptide Based on Amino Acids 27–39 of Serum Amyloid P Component Inhibits Lipopolysaccharide-Induced Responses in Human Blood. J. Immunol..

[216] Larrick J.W., Hirata M., Balint R.F., Lee J., Zhong J., Wright S.C. (1995). Human CAP18: A Novel Antimicrobial Lipopolysaccharide-Binding Protein. Infect. Immun..

[217] Al-Adwani S., Wallin C., Balhuizen M.D., Veldhuizen E.J.A., Coorens M., Landreh M., Végvári Á., Smith M.E., Qvarfordt I., Lindén A. (2020). Studies on Citrullinated LL-37: Detection in Human Airways, Antibacterial Effects and Biophysical Properties. Sci. Rep..

[218] Youn J.H., Kwak M.S., Wu J., Kim E.S., Ji Y., Min H.J., Yoo J.H., Choi J.E., Cho H.S., Shin J.S. (2011). Identification of Lipopolysaccharide-Binding Peptide Regions within HMGB1 and Their Effects on Subclinical Endotoxemia in a Mouse Model. Eur. J. Immunol..

[219] Ghiselli R., Giacometti A., Cirioni O., Circo R., Mocchegiani F., Skerlavaj B., D’Amato G., Scalise G., Zanetti M., Saba V. (2003). Neutralization of Endotoxin in Vitro and in Vivo by Bac7(1-35), a Proline-Rich Antibacterial Peptide. Shock.

[220] Zughaier S., Svoboda P., Pohl J. (2014). Structure-Dependent Immune Modulatory Activity of Protegrin-1 Analogs. Antibiotics.

[221] Xiao Y., Dai H., Bommineni Y.R., Soulages J.L., Gong Y.-X., Prakash O., Zhang G. (2006). Structure-Activity Relationships of Fowlicidin-1, a Cathelicidin Antimicrobial Peptide in Chicken. FEBS J..

[222] Matsuzaki K., Sugishita K., Miyajima K. (1999). Interactions of an Antimicrobial Peptide, Magainin 2, with Lipopolysaccharide-Containing Liposomes as a Model for Outer Membranes of Gram-Negative Bacteria. FEBS Lett..

[223] Nagaoka I., Yomogida S., Tamura H., Hirata M. (2004). Antibacterial Cathelicidin Peptide CAP11 Inhibits the Lipopolysaccharide (LPS)-Induced Suppression of Neutrophil Apoptosis by Blocking the Binding of LPS to Target Cells. Inflamm. Res..

[224] Wu J., Mu L., Zhuang L., Han Y., Liu T., Li J., Yang Y., Yang H., Wei L. (2015). A Cecropin-like Antimicrobial Peptide with Anti-Inflammatory Activity from the Black Fly Salivary Glands. Parasit. Vectors.

[225] Meadows C. (2018). Aseptic Sampling Best Practices Endotoxin Binding Affinity. Sartorius.

[226] Novitsky T. (1988). The Problems with Plastics. Lal Update.

[227] Péterfi Z., Kocsis B. (2000). Comparison of Blocking Agents for an ELISA for LPS. J. Immunoass..

[228] Kempler G., Ray B. (1978). Nature of Freezing Damage on the Lipopolysaccharide Molecule of *Escherichia coli* B. Cryobiology.

[229] Douwes J., Versloot P., Hollander A., Heederik D., Doekes G. (1995). Influence of Various Dust Sampling and Extraction Methods on the Measurement of Airborne Endotoxin. Appl. Environ. Microbiol..

[230] Bennett G.M., Seaver A., Calcott P.H. (1981). Effect of Defined Lipopolysaccharide Core Defects on Resistance of *Salmonella Typhimurium* to Freezing and Thawing and Other Stresses. Appl. Environ. Microbiol..

[231] Nguyen M.P., Tran L.V.H., Namgoong H., Kim Y.H. (2019). Applications of Different Solvents and Conditions for Differential Extraction of Lipopolysaccharide in Gram-Negative Bacteria. J. Microbiol..

[232] Smith P.K., Krohn R.I., Hermanson G.T., Mallia A.K., Gartner F.H., Provenzano M.D., Fujimoto E.K., Goeke N.M., Olson B.J., Klenk D.C. (1985). Measurement of Protein Using Bicinchoninic Acid. Anal. Biochem..

[233] Papadiamantis A.G., Klaessig F.C., Exner T.E., Hofer S., Hofstaetter N., Himly M., Williams M.A., Doganis P., Hoover M.D., Afantitis A. (2020). Metadata Stewardship in Nanosafety Research: Community-Driven Organisation of Metadata Schemas to Support FAIR Nanoscience Data. Nanomaterials.

[234] PubMed Keyword “Endotoxin” 5203 Events, Keyword “Lipopolysaccharide” 7853 Events. https://pubmed.ncbi.nlm.nih.gov/.

